# Do hippocampal pyramidal cells respond to nonspatial stimuli?

**DOI:** 10.1152/physrev.00014.2020

**Published:** 2021-02-16

**Authors:** John O’Keefe, Julija Krupic

**Affiliations:** ^1^Sainsbury Wellcome Centre and Department of Cell and Developmental Biology, University College London, London, United Kingdom; ^2^Department of Physiology, Development and Neuroscience, University of Cambridge, Cambridge, United Kingdom

**Keywords:** cognitive map theory, hippocampus, hippocampal units, memory, place cells

## Abstract

There are currently a number of theories of rodent hippocampal function. They fall into two major groups that differ in the role they impute to space in hippocampal information processing. On one hand, the cognitive map theory sees space as crucial and central, with other types of nonspatial information embedded in a primary spatial framework. On the other hand, most other theories see the function of the hippocampal formation as broader, treating all types of information as equivalent and concentrating on the processes carried out irrespective of the specific material being represented, stored, and manipulated. One crucial difference, therefore, is the extent to which theories see hippocampal pyramidal cells as representing nonspatial information independently of a spatial framework. Studies have reported the existence of single hippocampal unit responses to nonspatial stimuli, both to simple sensory inputs as well as to more complex stimuli such as objects, conspecifics, rewards, and time, and these findings been interpreted as evidence in favor of a broader hippocampal function. Alternatively, these nonspatial responses might actually be feature-in-place signals where the spatial nature of the response has been masked by the fact that the objects or features were only presented in one location or one spatial context. In this article, we argue that when tested in multiple locations, the hippocampal response to nonspatial stimuli is almost invariably dependent on the animal’s location. Looked at collectively, the data provide strong support for the cognitive map theory.

## Graphical abstract

**Figure:**
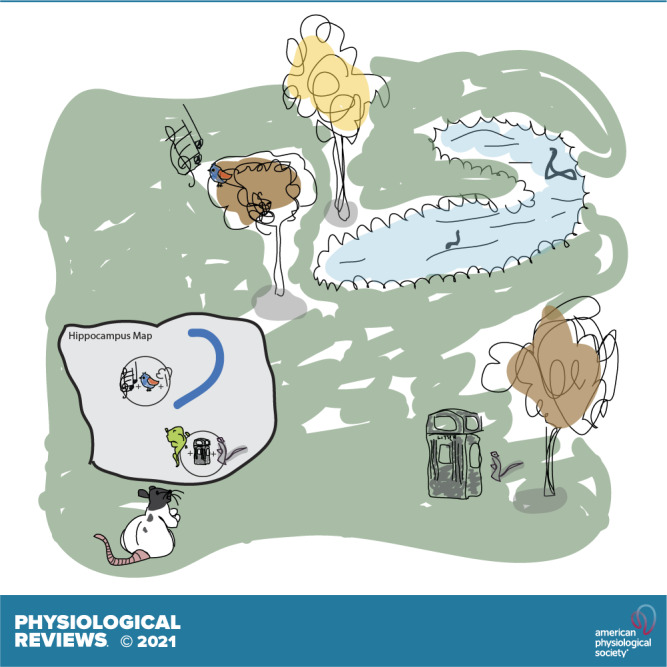

## 1. INTRODUCTION

The original formulation of the cognitive map theory specified that the hippocampus is part of a “memory system, which contains information about places in the organism’s environment, their spatial relations, *and the existence of specific objects in specific places*” (Ref. 1, p. 2; emphasis added). The basic idea was that the cognitive map consisted of a set of place representations together with the items, features, or objects that they contained and these representations were linked together by vectors specifying the direction and distance between them (2). Using this cognitive mapping system, an animal would know not only where it was in a familiar environment but what objects (including rewards and punishments) it should expect to find there and how to flexibly navigate to their locations. This idea was grounded in the finding that the very first place cell reported required a tactile stimulus in the preferred location to activate the cell. The same tactile stimulus elsewhere was ineffective (3). Subsequently it was clear that a percentage of place cells (called misplace cells) fired more when there was an object in the place field or when an object usually found there was missing (4). Thus, there are two types of place cell, simple place cells that fire whenever the animal goes to a particular location and more complex place cells, feature-in-place cells, that increase their firing in the place field when it contains an additional feature or when a feature that normally occupied that place is not there. This latter mismatch function was considered to be the basic process by which maps were originally built and subsequently modified when sensory inputs conflicted with stored representations (Ref. 1, p. 246–7).

Other formulations of hippocampal function (see, e.g., Refs. 5–10) usually see it as much broader than purely spatial and concentrate on the processes carried out there, such as the coding of sequences, irrespective of the specific material being stored and manipulated. Perhaps the clearest formulation of this position is that by Eichenbaum and Cohen. In their broader view, “… the hippocampus supports declarative memory by providing a general relational processing mechanism, across species and across the different tasks, modalities, and domains tested,” (11) and, more recently, “…The representational schemes that underlie relational processing of ongoing experiences are *1*) the representation of events as the relations among objects within the context in which they occur, *2*) the representation of episodes as the flow of events across time, and *3*) the interleaving of events and episodes into relational networks, supporting the ability to draw novel inferences from memory…,” (Ref. 8, p. 764). As can be seen in the second quotation, an important recent modification of the relational theory has been to incorporate the notion of context as a modulating aspect of experience, which, if context can be read as spatial context, brings it closer to the original cognitive map position.

In this article, we do not deal with the human hippocampus, which from the cognitive map point of view requires enhancement of the basic spatial framework by the addition of language and the metaphorical use of language [see writings on language (1, 12, 13)]. In the original formulation of the cognitive map theory the coding of time was reserved for the human hippocampus and was not postulated to be carried out by the infrahuman hippocampus. The present review of the literature on temporal coding makes it clear that the rodent hippocampus also incorporates a temporal signal, and this is dealt with in sect. 9.

The logic we are pursuing in this article is as follows: All theories agree that hippocampal pyramidal cells represent both spatial and nonspatial information. One important difference between cognitive map theory and the others lies in their predictions about the relationship between these two classes of information. Cognitive map theory states that the concept of “place” is generated in the hippocampus whereas nonspatial inputs are generated elsewhere and projected to the hippocampus, where they are embedded in place representations. Pure nonspatial responses independent of the animal’s locations may exist in the hippocampal formation as inputs from elsewhere, but even this remains unproven because, in general, experiments that have reported these have failed to test their dependence on the animal’s location. That is, the “pure nonspatial responses” are actually covert feature-in-place responses. Other theories, on the other hand, put spatial and nonspatial responses on an even footing and therefore predict that there will be some cells showing spatial responses independent of nonspatial responses, others showing nonspatial responses independent of spatial responses, and a third group with joint spatial/nonspatial responses. What these latter theories will find difficult to explain is the absence of purely nonspatial responses where these are adequately tested, i.e., by being presented in different locations.

We deal first with studies concerned with features such as single modality sensory inputs and subsequently with more complex stimuli such as objects, conspecifics, rewards, and time (TABLE 1). Although studies in the ventral hippocampus are far fewer than in the dorsal hippocampus, these are included and discussed where appropriate throughout the article. We end the article with some observations and suggestions for future studies. TABLE 1 includes information about whether the spatial context was taken into account in a study. It is our contention that studies that take location into account almost invariably find that the responses of hippocampal cells are strongly gated by spatial context. Earlier discussions of the role of spatial context in hippocampal function are Refs. 64–67.

**Table 1.** Summary of results on spatial dependence

Reference	Nonspatial Feature	Test	Result	Place Field, Goal Location, or Spatial Context Tested?	Place Field, Goal Location, or Spatial Context Dependent?
Wood et al. (14)	Odors	Continuous nonmatch odor test	Some pure odor responses, some place, some odor in place	Yes	Some but not all
Manns et al. (15)	Odors	Odor identity and recency	Weak identity of odors; no signal for recency or ordinal position; odor-in-place cells often change rate over trials	Yes	Yes
Komorowski et al. (16)	Odors	Odor/location conditional discrimination	Odor-in-place responses	Yes	Yes
MacDonald et al. (124)	Odors	Odor match go/no-go	CA1 cells signal odor identity	No	n/a
Igarashi et al. (17)	Odors	Odor/location association	dCA1 cells signal odor identity and phase lock to low-freq gamma LFP	No	n/a
Allen et al. (18)	Odors	Odor sequence go/no-go	Hippocampal cells detect odors out of usual sequence	No	n/a
Herzog et al. (19)	Taste	4 Tastes	Taste-in-place response in CA1 place field	Yes	Yes
Sakurai (20)	Tones	Pitch or duration discrimination	Both coded in CA1/3	No	n/a
Moita et al, (21)	Tone	Auditory-eyelid shock conditioning	Response in CA1 place field	Yes, in single box, place field dependent	Yes
Itskov et al. (22)	Complex tones	2 Tones, turn left; other 2 tones turn right; 2 locations	Tone in place specific	Yes, in 2 different places	Yes
Shan et al. (23)	Tone	Tone/eyeblink conditioning	Conditioned unit response place dependent	Yes	Yes
Aronov et al. (5)	Tone	Ascending pitch	Freq-specific CA1 cells	No	n/a
Itskov et al. (24)	Texture	2 Textures, turn left; other 2 right; 2 locations	Texture in place specific	Yes, in 2 different places	Yes
Zhao et al. (25)	Visual	CA1/3 VR place field created	Field follows Vis stimulus in 1 VR, not other	Yes	Yes
Manns and Eichenbaum (26)	Incidental object-in-place	Circular arena recognition task: objects in same locations for 3 laps and then moved to new locations and new objects added in new locations	No object-only cells	Yes	Yes
von Heimendahl et al. (27)	Conspecific/or object	CA1,2,3 sniffing objects and rats	Place fields modified by objects/rats	Yes	Yes
Zynyuk et al. (28)	Conspecific	2 Rats foraging	Slight deterioration in spatial signal	Yes	Yes
Alexander et al. (29)	Conspecific/or object	2 Rats foraging	Larger CA2 place fields, global remapping of place cells	Yes	Yes
Rao et al. (30)	Conspecific/or object	Rat reaching out across the gap between 2 platform and facially interacting with another rat	Ventral CA1, CA2 place fields modified by objects/rats	No	n/a
Danjo et al. (31)	Conspecific	Rat observes conspecific running on a T maze and chooses the arm not chosen by it	CA1 place fields modulated by observing rat	Yes	Yes
Omer et al. (32)	Conspecific/object	Conspecific location task: bat observes and mimics conspecific flying on a Y maze to get reward	CA1 place fields modified by bats/objects	No	n/a
Eichenbaum et al. (125)	Goal in approach olfactory discrimination	Discriminate between 2 odors and approach reward port	Hippocampal pyramidal cells respond during different aspects of task inc approaching reward area	No	n/a
Breese et al. 1989 (126)	5 Water goals on open platform	Change location of water goal during trial	Most place cells fire in 1 of 5 randomly rewarded locations but shift location to goal when only 1 rewarded	Yes	Yes
Wiener et al. (127)	Odor-goal approach, or place discrimination	Simultaneous odor discrimination or radial maze navigation in corners of same box	Cells fire during both tasks but fields different, probably because of task demands	No	n/a
Speakman and O’Keefe (128)	Goal	Goal reversal in +maze spatial task	No change in place fields	Yes	No
Markus et al. (129)	Goal	Open field foraging or navigate to goal	Fields more directional in navigation task	No	n/a
Gothard et al. (130)	Small moving box	Approach and exit movable box in open field and linear track	Moving box-related pyramidal cells are different depending on spatial context	Yes	Yes
Hollup et al. (131)	Goal	Annular water maze	Twice as many place fields in goal as elsewhere	No	n/a
Fyhn et al. (132)	Goal	Change platform location in annulus maze.	Approximately 1/3 of the recorded pyramidal cells fired exclusively at goal, 3 times any other bin	Yes	Yes
Kobayashi et al. (33)	Goal	Random foraging and then goal-directed behavior between 2 goals for brain stimulation reward	Some fields move to the goal after goal-directed behavior; no cells respond to both goals	Yes	Yes
Lee et al. (34)	Goal	Free running T-maze alternation	Field rates in stem alter with goal; movement of stem fields, but not elsewhere, toward goal	Yes	Yes
Ainge et al. (35)	Goal	4-Component maze with common start and segments but 4 different goals	No place cells fired in all 4 goals but differentially reflecting destination; others represented animal’s location in start arm and not destination	Yes	Yes
Hok et al. (36)	Goal	Go to a location in open field and wait for 2 s to get reward randomly scattered around the environment; secondary firing in goal for 84% of place cells	Goal firing after several hundred milliseconds in goal	No	n/a
Hok et al. (37)	Goal	As above	Goal firing after several hundred milliseconds in goal, longer for place task	No	n/a
Siegel et al. (38)	Goal	Approach and wait in unmarked zone in open field for 1 s for scattered reward	No firing in goal	No	n/a
Dupret et al. (39)	Goal	Several goal locations on hole board	Up to 20% goal cells in CA1, but not CA3, place fields were reorganized to represent new goal locations	Yes	Yes
Ainge et al. (40)	Goal	Conditional T maze with left/right choice dependent on flashing or stationary light	Firing rate of place cells in start/stem dependent on destination	Yes	Yes
McKenzie et al. (41)	Goal	Circular track with 20 wells, several baited at the same time; rat must stop for 5 s to get reward	Cells fired at some but not all goal locations during WAIT events	Yes	Yes
Grieves et al. (42)	Goal	Navigate to 3 different goals by 4 routes	Overlapping route sections but different place fields, only 4% goal cells	Yes	Yes
Hayashi et al. (43)	Goal	Place preference task, to unmarked goal	Increased firing rates in goal area in controls but not disc-1 mutants	No	n/a
Danielson et al. (44)	VR	Foraging and goal navigation in VR	Deep CA1 place cells remap more between tasks but more stable near goal; superficial CA1 cells more stable between tasks	Yes	Yes
Mamad et al. (133)	Goal	Flip-flop T maze pitting place vs. response learning	Remapping of cells on maze with a preference for the side of the chosen arm	Yes	Yes
Gauthier and Tank (45)	Goal	VR	5% of CA1 or subiculum cells show excess density of fields around the reward location in 2 different environments.	Yes	No
Kobayashi et al. (134)	Reward value	Open field navigation for brain stimulation reward	Rewarding stimulus activates place cell but only in place field	Yes	Yes
Lee et al. (46)	Reward value	Automatic T maze, unsignaled variation of probability of reward in each arm	Firing in 15% CA1 and 11% subiculum reflect the value of the reward and current and/or previous choice	No	n/a
Lee et al. (47)	Reward value	Probability of rewards signaled by different tones in goal	Difference in CA1 but not CA3 responses	No	n/a
Tryon et al. (48)	Reward value	Maze choice between predictable small reward or probabilistic large reward	Place field rates vary in goal during low-probability reward trials	No	n/a
Duvelle et al. (49)	Goal vs. reward value	2 Goals separate from reward location	No overrepresentation of goal; CA1/CA3 goal activity related to location or behavior but not value	Yes	Yes
Spiers et al. (50)	Goal direction	Distance to 4 goals	Firing rates increase with distance to goal	n/a	n/a
Sarel et al. (51)	Goal direction	Vector to goal	CA 1 cells relate to goal in bats	n/a	n/a
Aoki et al. (52)	Goal direction	Goals in 4 corners of box	Goal direction firing in small % (4%) of CA1 cells	Yes	Yes
					
Czurkó et al. (53)	Wheel cells	Running in wheel	Continuous firing during wheel running	Yes	Yes
Manns et al. (15)	Time	Odor identity and recency	Weak identity of odors; no signal for recency or ordinal position; odor-in-place cells often change rate over trials	Yes	Yes
Pastalkova et al. (54)	Time	Running in wheel in figure-8 maze	Short duration CA1 time cells	Yes; different patterns in nonmemory treadmill	Yes
Macdonald et al. (55)	Time	Odor match-to-sample task	Short duration CA1 time cells	Yes	Partly. Many time-in-place cells (73%) but 11% time-only cells
Mankin et al. (56)	Time in place	Repeated foraging in open field arenas	CA1/3 place cell patterns deviate over hours/days	Yes	Yes
Kraus et al. (57)	Time	Running on treadmill in figure-8 maze	Short duration CA1 time and distance cells	No	n/a
MacDonald et al. (124)	Time	Odor match go/no-go	CA1 cells fire at preferred time during delay period	No	n/a
Ziv et al. (58)	Time	Repeated exploration of an open field arena	CA1 place cell patterns change over time	No	n/a
Mankin et al. (59)	Time in place	Repeated exploration of open field arenas	CA2 place cell patterns deviate over hours/days	Yes	Yes
Kraus et al. (135)	Time	Running on treadmill in figure-8 maze	Short duration grid time and distance cells	No	n/a
Salz et al. (60)	Time	CA1/3 running on treadmill in figure-8 maze	Short duration CA1/3 time cells	No	n/a
Villette et al. (61)	Duration vs. distance	CA1 running on nonmotorized treadmill in dark	50% Code for distance run, 11% for duration, 39% both	No	n/a
Haimerl et al. (62)	Duration vs. distance	CA1 running on nonmotorized treadmill in dark	Cell correlates shift between duration and distance on consecutive days	No	n/a
Sun et al. (63)	Events independent of time or space	CA1 cells during laps on a 4-circuit track	CA1 cells signal lap number independently of place fields	Yes	No

LFP, local field potential; VR, virtual reality; dCA1, dorsal CA1; n/a, not applicable.

## 2. HIPPOCAMPAL SINGLE UNITS RESPOND TO SIMPLE SENSORY STIMULI

Since the pioneering studies in the late 1960s by Jim Olds and colleagues (e.g., Ref. 68), there has been considerable evidence of nonspatial responses of single units in the hippocampal formation to simple sensory inputs. The existence of such inputs might suggest that the hippocampus has a broader function than spatial; alternatively, these nonspatial responses might actually be signaling a feature in place, where the spatial nature of the response has been missed by the narrowness of the experimental design, resulting in the location-driven responses being masked by the fact that the features were only presented in one location or one environment. Indeed, careful examination of the details of these studies (TABLE 1) shows that, where tested, the hippocampal response to nonspatial stimuli is almost invariably dependent on the animal’s location. We look first at simple inputs in the five sensory modalities.

## 3. OLFACTORY AND TASTE STIMULI

A series of experiments on normal and hippocampal-lesioned animals have dissected the respective roles of the olfactory cues themselves and their spatial (and more recently temporal) context in successful performance on a variety of discrimination, inference, and memory tasks. In general, the hippocampus is not required for many olfactory tasks: simple discriminations when both cues are present at the same time (69, 70), recognition paradigms where the animal has to respond to novel but not to recently experienced odors (71), nonmatch to a previously presented sample (72). Simultaneous discrimination is, however, affected by lesions of the perirhinal cortex (73), and, somewhat surprisingly, lesions of the entorhinal cortex alone result in improved performance on go/no-go discriminations (74). The hippocampus itself becomes important in odor-sequence learning. In a study designed to demonstrate nonspatial sequence coding, Fortin and colleagues (75) presented rats with a sequence of 5 odors in the same place drawn at random from a library of 20 and asked them subsequently to identify which of a pair of the 5 was experienced earlier. Animals with hippocampal damage were severely impaired but could recognize the individual odors if paired with an unexperienced odor. But we would argue that location, even when not explicitly tested, is still an important factor in this class of studies. In a separate study designed to distinguish the contributions of spatial location and odor (76), normal animals were trained on the sequence task, where each different odor was presented in a different location and subsequently spatial location vs. odor alone probes given: pots containing the odors were presented in new locations or in their correct locations but without the odors. The animals initially approached the correct location 69% of the time but if this did not contain the correct odor switched to the other pot, resulting in an overall performance of 76% correct. In further tests, location by itself proved incapable of supporting correct choice, whereas odors by themselves when presented in new locations resulted in performance equal to that during the standard odor-in-place task. Animals with subsequent hippocampal lesions failed completely on the standard odor-in-place sequence task, showed normal performance on the odor-alone sequence probe, and showed significantly below-chance performance on the location-alone sequence probe. These results show that this and presumably similar recency discrimination tasks that appear simple on the surface are actually more complex. Although hippocampal-lesioned animals can discriminate the odors and know which one was experienced earlier, they do not use this information by itself to solve the what-when task. On this interpretation, the hippocampal system codes for the temporal ordering of the odor-in-place experiences and this knowledge takes preference over the simpler odor strength system. An early influential study by Wood et al. (14) recorded hippocampal pyramidal neurons during a variant of a continuous olfactory recognition task. Rats were trained on an open platform to approach a small cup containing sand scented with one of nine odors. On each trial, the cup was placed first in one of nine locations and then in another. If a cup had a different odor on the second placement from that of the previous one, it contained food the animal could dig for (nonmatch); if the odor was the same, there was no food (match). The behavioral paradigm allowed the dissociation of odor identity, location, and the match/mismatch aspects of the task. Ten cells of 127 (7.8%) responded to odor in the absence of any other correlate, 14 (11%) solely to location, and 13 (10.2%) solely to the match/mismatch aspect of the task. In all, 20% of cells had nonspatial correlates and 32% took location into account. Interestingly, the two cells that had the best odor selectivity both showed strong significant interactions with location. Unlike some of the other olfactory tasks we have discussed above, which are sensitive to hippocampal damage, a similar task has been shown by the authors of the Wood et al. study themselves (71) not to be disrupted by selective hippocampal damage; in fact, lesioned animals were able to remember and ignore up to 24 previously experienced odors at a time, a number comparable to control animals.

**FIGURE 1.**
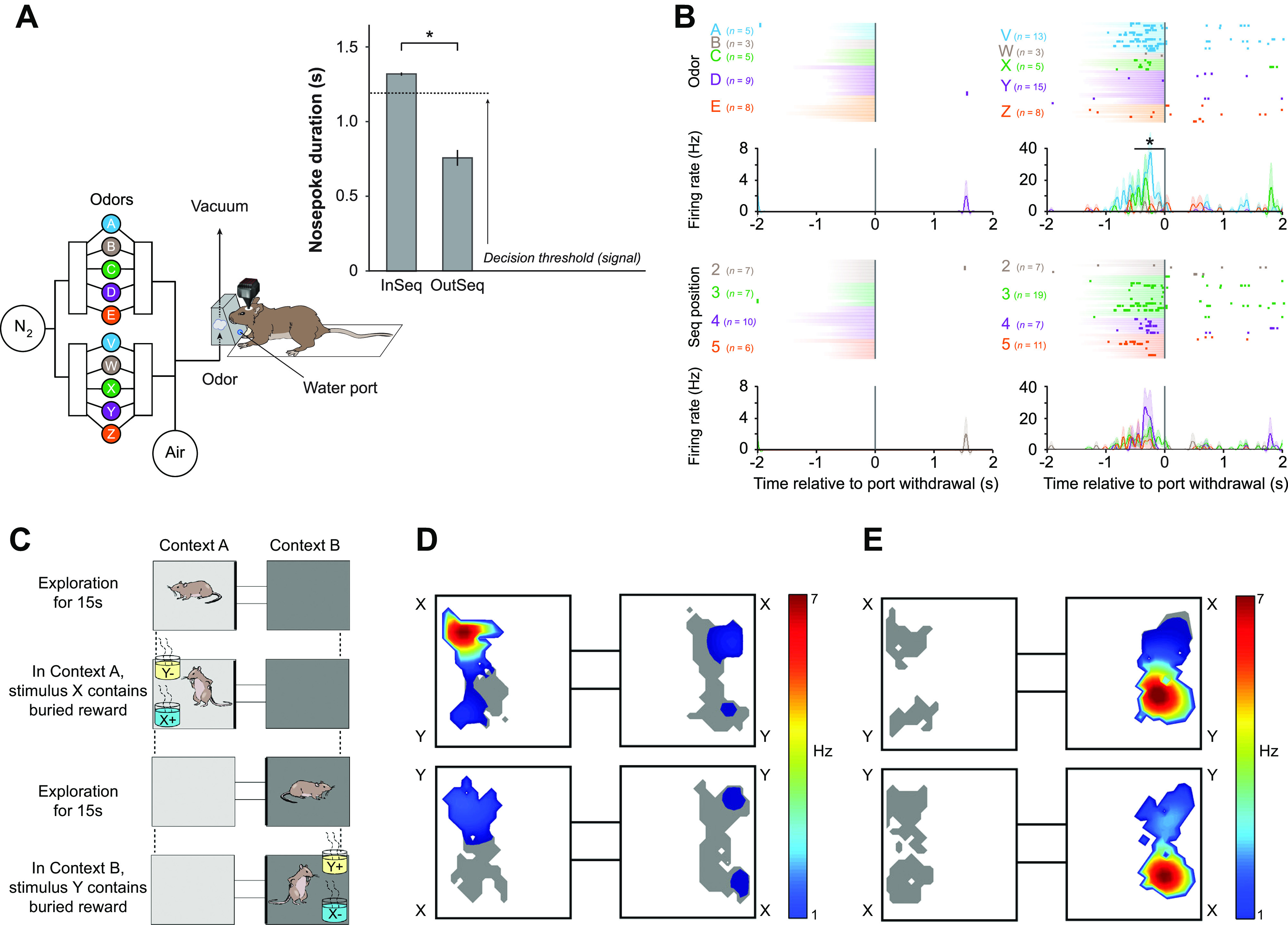
Olfactory responses of hippocampal pyramidal cells in an olfactory spatial conditional discrimination are gated by the spatial context. *A*: sequence memory task design and performance. With an automated odor delivery system (*left*), rats were presented with a sequence of 5 odors delivered in the same odor port. In each session, the same set of odors was presented multiple times with all items in sequence (ABCDE) or out of sequence (e.g., ABDDE). Each odor presentation was initiated by a nose poke, and rats were required to correctly identify the odor as either in sequence (by holding their nose poke response until a signal at 1.2 s) or out of sequence (by withdrawing their nose poke before the signal) to receive a water reward. Performance shown at *right*. *B*: an example of a conjunctive sequence cell showing selectivity for specific conjunctions of item (*top*) and sequence position information (*bottom*). *C*: schema of the Komorowski et al. (16) experiments: to get the reward a rat had to choose odor X in context A and odor Y in context B. *D*: an example of an odor-in-place cell recorded during the spatial conditional discrimination task. Place field heat map shows cell fired when the animal sniffed at the X odor in the right-hand position in box A but not under any of the other conditions. See text for more details. *E*: a classic place cell responded to a location in box B irrespective of the odor there. *A* and *B* from Ref. 18, with permission from *Journal of Neuroscience*; *C–E* from Ref. 16, with permission from *Journal of Neuroscience*.

In a modified odor sequence memory test, Allen and colleagues (18) reported that 11% of hippocampal cells respond differentially to correct and incorrect sequences of odors, and this number correlated with task performance (FIGURE 1*A*); of these, 40% showed selectivity for specific conjunctions of item and sequence position (FIGURE 1*B*). Rats first learned a sequence of five odors. On a probe trial, some of the odors were misplaced within the sequence and the rats were asked to hold the nose poke response if an odor was presented in sequence or withdraw if out of sequence. Importantly, in this test the location of the animal was not changed, so it is not possible to exclude that these were place-dependent responses.

Two important studies from Eichenbaum’s laboratory did include this control and, we would argue, have provided some of the best evidence for an odor-in-place role for hippocampal pyramidal cells. The first (16) was an odor/spatial context conditional discrimination task in which the animal had to dig for a reward in one of two odor pots in one context, arena A, and in the other pot in a second context, arena B (FIGURE 1*C*). The two arenas were connected by a passageway so the rat could shuttle between them. The same pair of scented digging pots were placed, in turn, into each arena, and the two locations in each arena were randomized to prevent the use of left/right egocentric spatial strategies. Thus the same olfactory stimulus could appear in four different locations, giving a total of eight olfactory-location combinations. In contrast to the Wood study, only 1 of 52 neurons fired differentially to the two odors irrespective of location. Odor-in-place cells comprised 31% of the cells, and pure place cells represented another 47%. The typical firing pattern of an odor-in-place cell (FIGURE 1*D*) makes it clear that recording from one stimulus in one location/context (FIGURE 1*D*, *top left*) would lead to the conclusion that this cell responded to odor X and not odor Y, and it is only the other conditions that reveal the roles of place and context. A simple place cell that did not conjunctively represent one of the odors is shown in FIGURE 1*E*. Even for this simple place cell, one cannot rule out the possibility that other nontested odors might have modulated the firing in the active location. Interestingly, the number of odor-in-place cells increased dramatically over the course of learning, rising from 6% of the cells at the beginning of learning to 31% at the end, and the increase correlated with the learning of the task. In contrast, the proportion of place cells did not change over the course of learning, nonsignificantly going from 47% at the beginning to 38% at the end of learning. Detailed analysis of these changes showed that the new odor-in-place cells developed from simple place cells and that the new simple place cells developed from previously nonresponsive cells. No new simple odor cells were reported as a result of learning. Our interpretation of this important set of results is that most or all CA1/CA3 pyramidal cells have the potential to become place cells and that many place cells have the potential to become odor-in-place cells or, more generally, feature-in place cells. This latter can occur under normal circumstances but may be accelerated during learning tasks that call attention to particular features in particular places. It is a shame that the experiment did not involve a control condition in which the same odors were rewarded in both places to test the role of context contingencies as a factor in the results. Hippocampal feature-in-place cells occur in experimental paradigms where the animal is not rewarded for making the association (19), or even when it is rewarded for paying attention to other aspects of the experiment (Ref. 26; see below). Simple odor cell responses do not appear to exist in the hippocampus before or after training on an odor/place association task. Another study from the Eichenbaum laboratory (15) also found a strong dependence of olfactory inputs on the animal’s spatial location and found evidence that this odor-in-location cue varied in strength across the testing period, making it a potential candidate for use in recency discriminations (see sect. 9).

Another clear example where taking the animal’s location into account changes one’s interpretation of hippocampal responses to sensory stimuli involved taste stimuli. There is good behavioral evidence that the hippocampus is involved in regulating food intake and that this might be due to the inhibition of feeding after an animal has eaten food. Clifton et al. (77) showed that the hippocampus is involved in the patterning of meals but not in the total amount of food ingested: animals with hippocampal lesions ate more often but took smaller meals than control animals and consequently did not gain weight. Henderson and colleagues (78) found that postprandial inactivation of the dorsal hippocampus resulted in a quicker return to eating after the last meal. The reason for this was speculated to be a deficiency in memory for the last meal (79). Herzog and colleagues (19) studied the response to different-tasting fluids, sweet (saccharine), salty (sodium chloride), neutral (distilled water), and bitter (quinine), injected directly into the mouths of rats foraging in an open field. The primary correlate of the vast majority of CA1 pyramidal cells (90%) was the animal’s location, and in addition 15% of these were responsive to tastes but only when the fluid was delivered in the cell’s place field. A much larger percentage of interneurons, 76%, also had taste responses. Place cells with taste responses had 23% larger place fields than those without, and there was a negative correlation between the spatial information content and the magnitude of the taste response. Taking both place cells and interneurons together, increased firing to injected fluids provided information about the presence of a taste at short latencies (80%) and the identity of the taste (37%) and its palatability (19%) at longer latencies, with some cells providing information about two or all three of these categories. Given these findings, it would be interesting to test whether the hippocampal inhibition of postprandial eating was location specific.

## 4. AUDITORY RESPONSES

**FIGURE 2.**
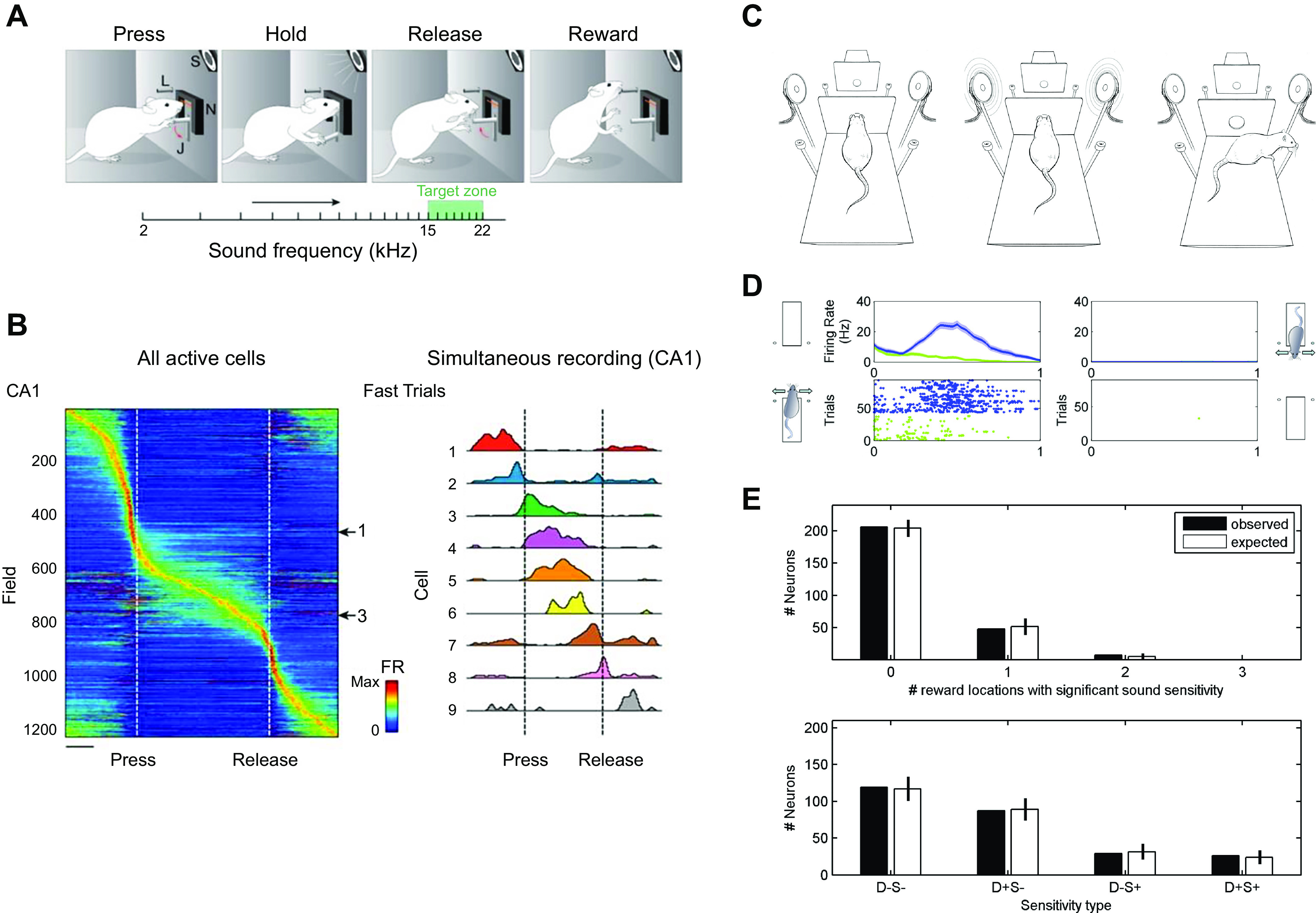
Acoustic hippocampal responses are gated by spatial context. *A*: schematic of the Aronov et al. (5) task: the rat had to hold down a lever to turn on a sound consisting of a frequency ramp ascending from 2 to 22 kHz, and then release the lever when the frequency reached a window of 15–22 kHz to get the reward. *B*: responses of all (*left*) and selected (*right*) cells during the auditory go/no-go frequency discrimination task. Some cells fire selectively to specific tones. *C*: schematic of the Itskov et al. (22) auditory discrimination task, in which there are 2 platforms in different locations facing in different directions. The same auditory stimuli are presented in different locations. *D*: hippocampal cells only respond to specific auditory stimuli when the animal is on one platform (*left*) but not the other (*right*). *E*: the actual and predicted distribution of cell responses if cell responses to the stimuli were different on the 2 platforms. *A* and *B* from Ref. 5, with permission from *Nature*; *C–E* from Ref. 22, with permission from *Journal of Neurophysiology*.

We found five studies that used auditory stimuli. Again, the pattern is the same as for olfactory stimuli. When location is ignored, hippocampal cells appear to respond to simple auditory inputs. Three studies took the location of the stimulus into account, whereas two did not (TABLE 1). A good example of the latter is the study of Aronov and colleagues (5); they trained rats on a simple go/no-go auditory discrimination in which animals had first to hold down a lever to turn on a sound consisting of a frequency ramp ascending from 2 to 22 kHz and then to release the lever when the frequency reached a window of 15–22 kHz to get a reward (FIGURE 2*A*). The speed of the frequency increase varied from trial to trial, ruling out the use of temporal coding. Both hippocampal and medial entorhinal cortex (mEC) cells were recorded, and 40% of CA1 cells were engaged at different points in the task, with 5.5% frequency aligned, i.e., responding at a particular point in the frequency ramp rather than more broadly or during behaviors before or after the ramp (FIGURE 2*B*). The comparable number for mEC cells was 9.4%. Across the population, all frequencies were represented in both regions despite the fact that the animal only had to identify frequencies within the target range. Most of the same cells did not respond during passive reproduction of the frequency ramp but tended to respond if the (passive) ramp was followed by a reward, although to a lesser extent than during the active task. There was some overlap between cells responding in this task and those with spatial fields tested in another environment. The authors conclude that hippocampal pyramidal neurons and mEC2/3 cells code for continuous nonspatial variables such as tones of different frequencies in a manner comparable to spatial variables such as location. However, similar to the odor sequence memory task of Allen et al. (18), they did not record from the same tone cells either in different parts of the same box or in different boxes, and therefore we cannot rule out the possibility that these were in fact tone-in-place cells, as was observed with odor-in-place cell responses. Another study of auditory responses in hippocampal pyramidal cells that also did not present the stimuli in different locations (20) found responses to tone stimuli.

A study that did take into account the context dependence of auditory responses was that in Ref. 22 (FIGURE 2*C*). Itskov and colleagues trained rats to discriminate between four auditory “vowels” or complex sounds while on two different platforms located in different parts of the same laboratory. Two of the sounds required the animal to turn left on each platform to get a reward and the other two to turn right. Platforms were oriented at 180° to each other, so these two similar egocentric responses required turning in opposite allocentric directions. Twenty-one percent of hippocampal pyramids had significant responses to at least one of the sounds on at least one of the platforms (FIGURE 2*D*). When the authors looked at the interaction between responses to a sound and the animal’s location they found that 87% of cells responded on only one of the four platform-response combinations, 13% on two, and none on more than two (FIGURE 2*E*). They concluded that this was exactly what one would expect if “the presence in a given neuron of a sound identity code for any one of the four tested reward spout locations does not predict whether the same neuron will discriminate between sounds for other response directions” (Ref. 22, p. 1828) and “a neuron’s tendency to differentiate two sound stimuli at one location in space did not predict whether the neuron would differentiate the same sounds at a different location” (Ref. 22, p. 1830).

Historically, one set of experiments particularly difficult for the cognitive map theory to explain has been the response of hippocampal cells to the presentation of an auditory conditioned stimulus (CS) in Pavlovian nictitating membrane conditioning experiments. In these experiments the animal is restrained in one place and CS/unconditioned stimulus (US) conditioning is carried out there. Lesion studies suggested that only when a delay was interposed between CS and US (trace conditioning) was an intact hippocampus necessary for this learning. In previous discussions of these findings (80, 81), we suggested that the responsive cells might actually be place cells whose fields coincide with the location of the testing box and the responses to the CS were similar to the CS-in-a-place responses reported by Moita et al. (21) in the rat. The latter recorded the responsiveness of complex-spike and theta cells to an auditory stimulus before and after delay classical conditioning in which the stimulus was paired with a brief electric shock to the eyelid. No complex-spike cells responded with a short latency to the tone before conditioning, but many did so after conditioning. However, they did so only if the animal was located in the place field of the cell during CS presentation. Shan and colleagues (23) studied place cells during eyeblink conditioning in rats running on a linear track, and their results go a long way to solving the conundrum. They conditioned eyeblink to a tone presented randomly as the animal ran on the track for a food reward. A considerable proportion (63%) of CA1 pyramids had place fields along the track or in the end boxes where food was delivered. Some of these fired to the CS but only when the animal was sitting quietly in the place field, exhibiting sharp wave ripples (SWRs) in the hippocampal local field potential. The unit response was equivalent to the level of place field activity observed when the animal ran through that part of the track. Unit activity outside the place field was inhibited by the CS. They conclude that “the most parsimonious explanation of the experimental data is that dorsal CA1 neurons are all place cells and the apparent responses to the non-spatial stimuli are due to an arousal-mediated resumption of place-specific firing” (23). This has the broader implication that place field firing is partly dependent on the ongoing level of arousal and is suppressed during SWRs, a state of low arousal.

## 5. TACTILE AND VISUAL RESPONSES

The Diamond group (24) also used the same four stimuli/two responses in two different locations paradigm reported above for auditory stimuli to test whether hippocampal neurons respond to texture independently of location. Similar to the auditory results, 86% of neurons encoded texture on just one platform, failing to distinguish between the same textures when the rat was positioned on the opposite platform. Moreover, among 21 neurons that encoded a single texture pair on each platform, only 6 demonstrated consistent “texture tuning”; the remaining 15 either discriminated between the same texture pair on both platforms but switched preference within the pair or else discriminated between different texture pairs on the two platforms. Texture was represented in the hippocampal response but only in conjunction with the context, and texture representations “remapped” when the animal performed the same task to the same tactile stimuli in different locations. “…In conclusion, texture in hippocampus was represented in conjunction with the context, with no relationship to the physical features of the stimuli” (24).

The visual system provides a strong exteroceptive sensory input to the hippocampal place, grid, and head direction cells that controls their spatial firing and is used to locate the animal in a familiar environment. For example, rotating a single visual cue card on the wall of the testing box rotates these spatial cells by the same amount (82, 83). When pitted against other cues such as those of the path integration system, visual cues strongly control the location of place fields (84) as long as the reliability of these visual cues is not devalued by moving them in the animal’s presence (85). So one might expect cells in the hippocampal formation to respond to visual cues. But are these responses dependent on the context? Zhao et al. (25) recorded intracellularly from CA1 pyramidal cells as the animals ran in an impoverished visual virtual reality environment. Replicating previous work, they found that they could create place fields in silent pyramidal cells by intracellular depolarization sufficiently strong to produce plateau potentials and fire the cell at a particular location in the virtual environment. Plateau potentials are long-lasting (hundreds of milliseconds) depolarizations in CA1 pyramidal neurons most likely normally generated by the activation of extrasynaptic *N*-methyl-d-aspartate receptors (NMDARs) (86).The artificially created place cells followed the movement of local visual cues within the same environment but totally failed to respond to the same cues when the virtual reality environment was changed from an oval to a triangular track. Similar findings were found in naturally occurring extracellularly recorded CA3 cells, suggesting that the CA1 responses could be inherited from there. Even powerful visual stimuli that could be shown to drive place cells in one environment failed to do so in another.

## 6. OBJECT RESPONSES

Several studies have looked at the response of hippocampal cells to objects. There is an extensive literature on object recognition and its neural substrates. The cognitive map theory postulated that there are two brain mechanisms for responding to novel objects. In the first, the response is determined by the familiarity of the object, which depends ultimately on recency of experience with the object and decays reasonably rapidly as a function of time. “The important variables controlling reactions to noticeable stimuli appear to be the recency and frequency of the same or similar experiences, the rate of change, and good or bad associations. On the present view these context independent reactions are a function of the extra-hippocampal taxon systems.” (Ref. 1, p. 242). This neural system is located outside the hippocampal cognitive map and therefore should be intact in animals with damage to the hippocampus. The extrahippocampal taxon brain region involved in novel object recognition has been identified as the perirhinal cortex (87).

The second system for detecting novel objects sits squarely in the hippocampal cognitive map, performing one of its core functions: the role of exploration of novelty for originally building and subsequently updating of the map. “Novelty, then, would seem to depend on a long-term memory sensitive to contextual (typically spatial) configurations and capable of remembering single occurrences. Within the framework of the present theory an item or place is novel if it does not have a representation in the locale system and thus excites the mismatch cells in that system.” (Ref. 1, p. 241). The theory assumes that exploration of novel objects is triggered by a mismatch between what the current representation within the hippocampus predicts will be found in a particular location and what is actually found there. This idea of a dual mechanism for detecting novelty has also been espoused by other authors (see, e.g., Refs. 88, 89).

One widely used paradigm, the novel object recognition (NOR) test, asks whether rodents can remember that they have recently experienced an object or that they have experienced one object more recently than one or several others. The standard NOR experiment consists in exposing the animal to one or more objects in specific locations in a specific box (the context) and then subsequently testing whether they remember this experience by replacing one or more objects with new ones. Rodents have a strong tendency to explore novel objects more than familiar ones, and this can be used as a measure of object familiarity, although there is little research designed to test whether the amount of exploration is a good measure of memory strength (see Ref. 90). The effects of lesions of the hippocampus on NOR have recently been reviewed (91). The authors found that, regardless of how the lesions were made, only between 28% and 33% of experiments found deficits. In one interesting experiment (92), rats were impaired if the hippocampus was lesioned after training but were subsequently unimpaired when they were later presented with a new test using novel objects. These results suggest that extrahippocampal areas can support object recognition but only if the hippocampus does not participate in encoding the original encounter with the object. The simplest interpretation is that two memory systems underlie object recognition, a hippocampal system subserving long-term context-dependent object-in-place memory and a perirhinal system providing short-term context-independent familiarity-based object memory. Priority is normally given to the hippocampal system, but if it is unavailable the second system is brought into operation.

**FIGURE 3.**
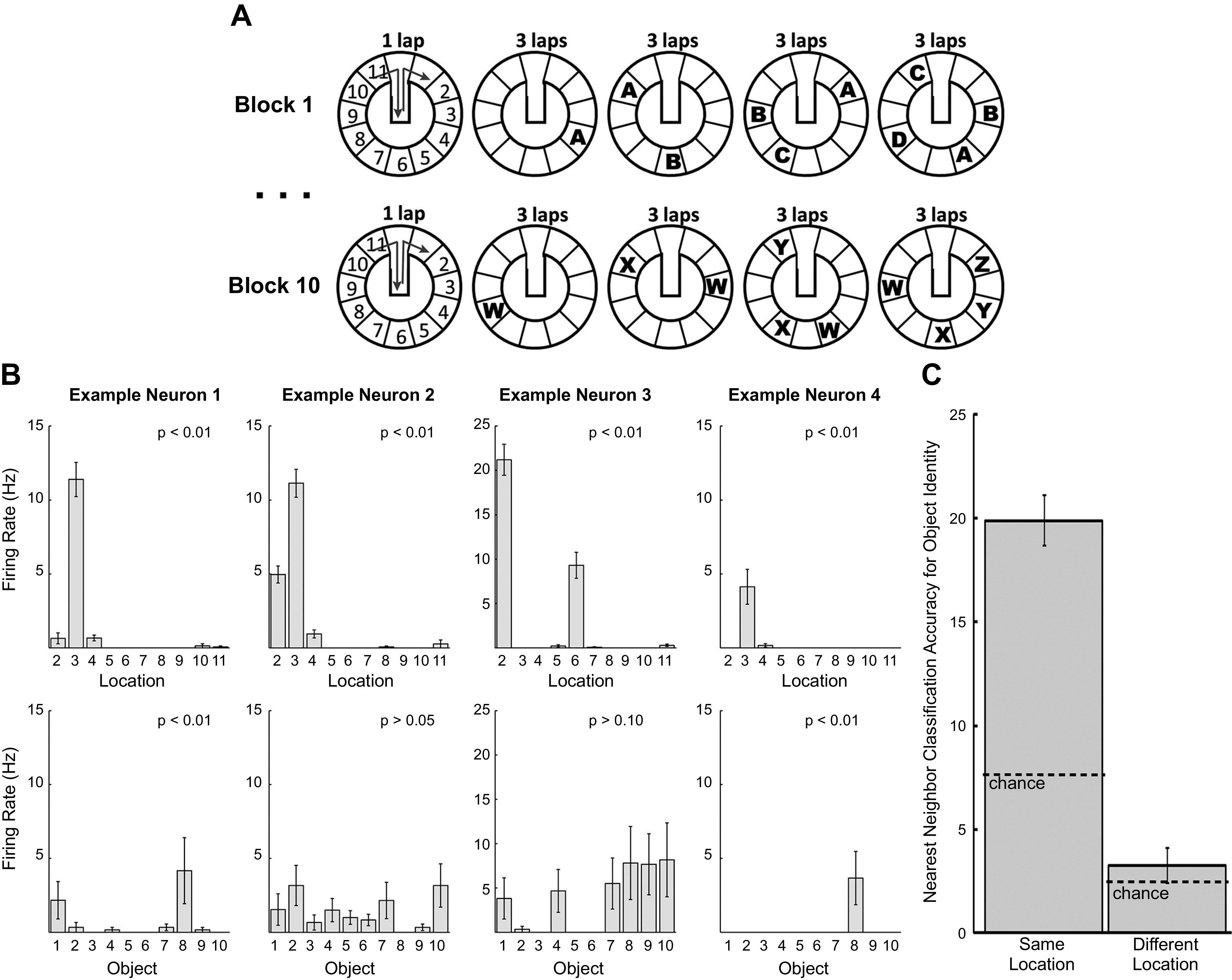
Hippocampal responses to objects. *A*: schematic of the Manns and Eichenbaum (26) running recognition task: rats circumnavigated an annular track for food reward after each lap. An item (e.g., A) was originally placed in a specific location on the track for 3 laps and then moved to a new location and an additional item (e.g., B) added in a new location for another 3 laps. During each block of 4 × 3 laps, 4 different objects were encountered in at least 2 different places. After this block, a new set of items was used and the procedure repeated for a total of 10 blocks and 40 different items. *B*: activity of hippocampal pyramidal cells in this task reflects location (*top*, all 4 cells) and to a lesser extent object identity (*bottom*, leftmost and rightmost cells). *C*: hippocampal cells distinguish between different objects but only when these objects are encountered in the same location on the running track. When objects are presented in different locations (*right*), hippocampal patterns do not distinguish between the same or different objects. When the objects are presented in the same location (*left*), the firing patterns do distinguish between them. From Ref. 26, with permission from *Learning & Memory*.

As we shall see, single-unit experiments strongly confirm this model and place it on a secure neural basis. The standard design of object recognition tasks does not lend itself to single-unit recording experiments, since they are usually one-shot events and would not provide enough cell data for statistical purposes. An experiment that successfully studied both object recognition and, independently, object-in-place recognition, was carried out by Manns and Eichenbaum (26) (FIGURE 3*A*). In an incidental recognition paradigm, rats ran around an annular track for a reward, encountering objects located at 12 different locations around the track of which a maximum of 10 were occupied during any individual trial. A total of 40 different objects were used during the course of the experiment. On every three successive laps, new objects were added and old objects were moved to new positions, allowing item memory to be dissociated from item-in-place memory. Decreased exploration for repeated objects and repeated places reflected memory for each of these independently. Behavioral analysis showed that the animals recognized the objects and also the location in which they occurred. Neural analysis showed that 60% of cells responded to the location of the objects and, in addition, 17% signaled the identity of the objects (see FIGURE 3*B* for typical examples). Most, if not all, of the identification of objects was due to the identification of that object in a particular location. Similar to the behavioral recognition data, the single-unit population response was more similar when the same rather than a different object was reexperienced in the same place but decreased markedly when that object was moved to a new place. (FIGURE 3*C*). It is clear from this experiment that the ability of hippocampal cells to distinguish objects from each other depends substantially on object-in-place recognition memory.

## 7. CONSPECIFIC RESPONSES

Several studies have looked at whether hippocampal pyramidal cells respond to conspecifics, using the response to objects as a control. They suggest that dorsal CA2 and ventral hippocampal cells show such responses. Rao et al. (30) used a “gap paradigm” that consists of two elevated platforms separated by a gap, with a rat on each platform spontaneously reaching out across the gap and performing facial interactions. Around 10% of ventral hippocampal cells showed strong firing modulation by the conspecific, especially in males, where cells were highly selective for females. The cells did not show such strong modulation by objects.

Dorsal CA2 cells, the main hippocampal target for vasopressin (the neurohormone involved in social behavior) and oxytocin (93), were also recorded in this study. These did not differentiate between the sexes of the conspecifics and never showed such a dramatic increase in activity as the ventral cells. Similar to ventral CA1–CA3 regions (94–97), it has been reported that dorsal CA2 place cells have significantly larger fields compared with dorsal hippocampal CA1 place cells, which may signal the context the animal occupies rather than a location per se, with a large percentage of dorsal CA2 place fields remapping in response to the introduction of a novel or familiar conspecific but none representing conspecifics per se (N.B. the same effect was found upon the introduction of a novel object) (29, 98). The Siegelbaum laboratory (99) has suggested that selective damage to dorsal CA2 pyramidal cells does result in alterations in social functions, perhaps via this remapping effect.

**FIGURE 4.**
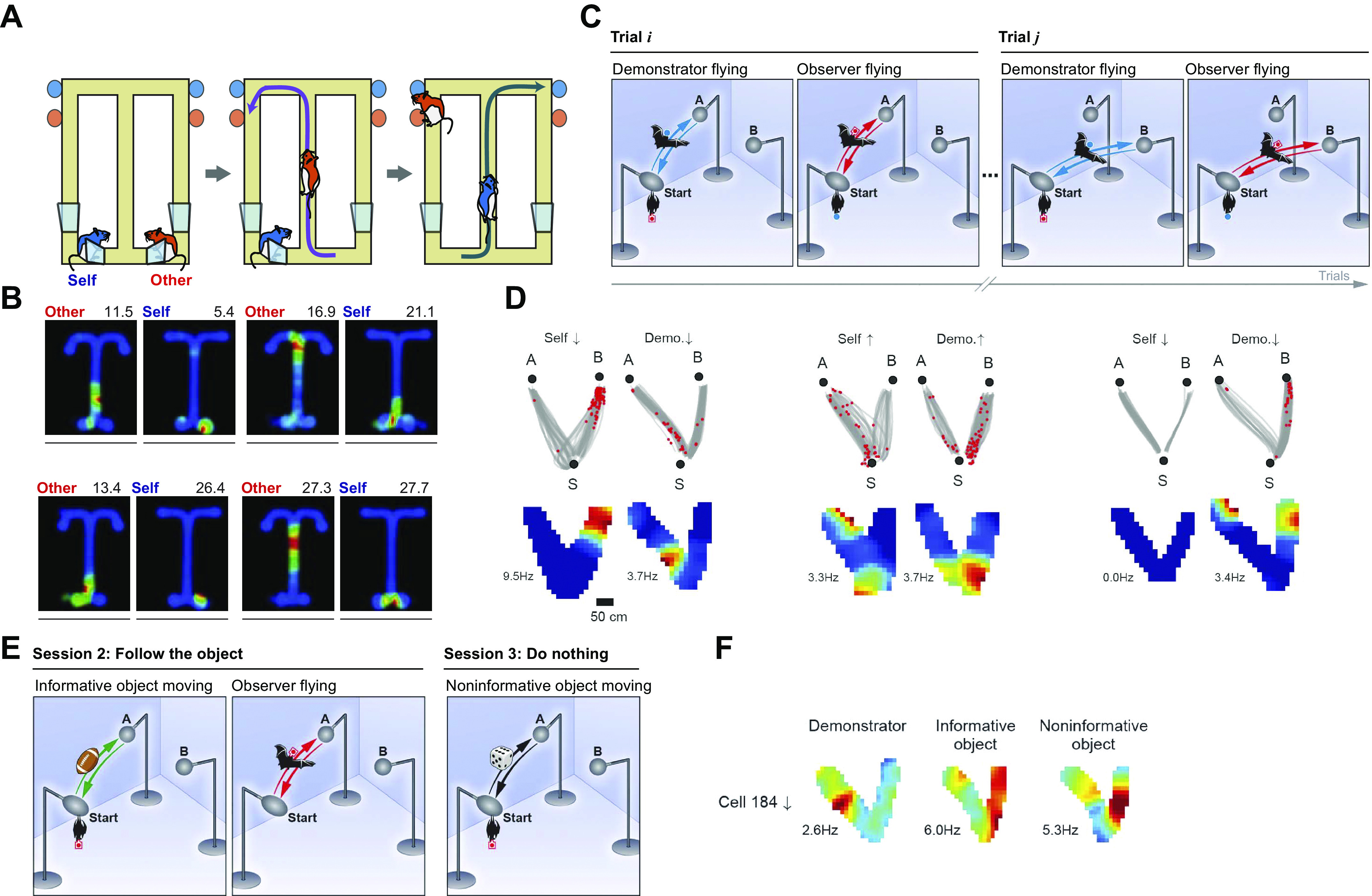
Hippocampal responses to conspecifics and goals. *A*: schematic of the rat conspecific location task: the subject rat had to observe a conspecific running on a figure-8 maze to choose the arm not chosen by the latter to obtain the reward. *B*: examples of 4 place cells whose firing was modulated by the location of the other rat in addition to the subject rat’s own location. *Left*: place field of the (other) conspecific rat’s location. *Right*: place field of the observer rat’s own location. *C*: schematic of the bat conspecific location task: the subject bat observed a conspecific flying to and from 1 of 2 goals A and B from start position S. *D*: 3 hippocampal cells that had social place fields. The left 2 cells fired in specific locations, both when the observer bat flew itself (*left*) or watched the flight of the demonstrator (*right*). The rightmost cell only fired to the flight of the demonstrator and not to the observer bat’s own flight. *E*: schematic of the bat object location task: the subject bat observed an inanimate object that it had to emulate (*center*) or not (*right*) moving to and from 1 of 2 goals A and B from start position S in addition to a conspecific (*left*). *F*: this cell fired in all 3 circumstances, whereas other cells represented only the objects or only the conspecific. Cells representing only the objects were often bidirectional, firing in both flight directions, whereas conspecific cells were usually unidirectional *A* and *B* from Ref. 31, with permission from *Science*; *C–F* from Ref. 32, with permission from *Science*.

There is little support for the idea that there are pure rat-specific cells in the dorsal hippocampus independent of the animal’s location. Instead it has been shown that the firing rate of hippocampal place fields is modulated by the presence of another rat: while Danjo and colleagues (31) reported as many as 85% (FIGURE 4, *A* and *B*), von Heimendahl et al. (27) only found 7% (not significant) of such CA1 place cells. The striking difference may be due to the attentional demands of the task. In the former study the subject rats had to observe the conspecifics to solve the T-maze task (FIGURE 4*A*), whereas in the latter experiment rats were simply interacting without any task. When objects were substituted for the conspecifics (27), if anything there was a slightly stronger object-in-place response. A similar finding was reported by Zynyuk and colleagues (28). In the bat CA1, Omer et al. (32) reported that 18% of pyramidal cells responded to the positions of a demonstrator conspecific on an airborne version of a Y-maze task (FIGURE 4, *C* and *D*). Similar to Danjo et al. (31), the subject had to observe the conspecific to be able to subsequently choose the correct arm on the Y maze. Fifty-seven percent of these cells also had self-place fields during navigation (FIGURE 4*D*).

However, the authors did not vary the location of the sessile observer bat during the other-bat observation phase task, making it impossible to know whether the remaining cells truly represent the position of a conspecific or, similarly to previous studies in rats, they were conjunctive place cells representing the subject bat’s current location modulated by another conspecific’s position on the Y maze. Also, in line with previous studies, many of the place cells (65%) that responded to conspecifics also responded to moving inanimate objects (FIGURE 4, *E* and *F*), although the authors reported some differences, e.g., they were bidirectional for moving objects but not for conspecifics (however, note that the objects but not the bats looked similar during both moving directions).

## 8. REWARD/GOAL CELLS

If one of the functions of the cognitive map is to provide information about the location of rewards and punishments so that the animal can learn to approach or avoid these places, then these places should be specially marked in the activity of place cells. When an animal forages in an open field without any definite goal, the hippocampal place fields usually present as omnidirectional, firing in all directions and tiling the surface of the environment with equal probability. Once a goal is introduced, the hippocampus and the animal’s behavior become focused on the goal. Studies reviewed (TABLE 1) point to four different ways in which goals might be represented in the hippocampus or have an influence on hippocampal place cells: *1*) overrepresentation of the goal location via fields moving toward the goal or new fields emerging; *2*) out-of-goal-location place cell firing modulation depending on whether the animal is heading toward the goal; *3*) dedicated goal cells; or *4*) goal-direction and -distance cells. Additionally, the value of the reward as well as time spent in the reward location may be reflected in the activity of hippocampal cells. We deal with each in turn. Importantly, it must be noted that introduction of a goal often significantly changes an animal’s behavior, resulting in strong directional, locational, and velocity sampling biases that can make experimental design and data interpretation challenging.

Place fields might cluster or fire more around the goal/reward location. Several studies show goals overrepresented, but this usually differed for different goals.

**FIGURE 5.**
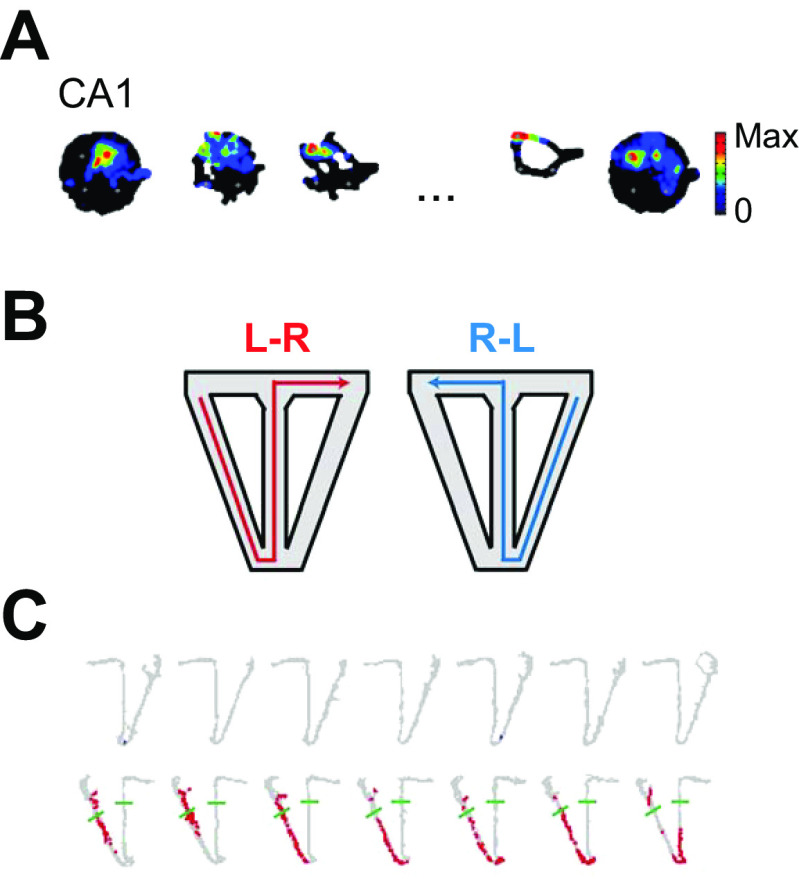
Movement of place cells toward goal. *A*: place field in the open field shifts toward goal location (pretraining field on *left*; posttraining on *right*) after training on 3 new goals (several training trials of a series in *center*). *B*: schematic of continuous T-maze alternation task. *Left*: path on return from previous left goal and run on stem prior to right goal choice (L-R, in red). *Right*: path on return from previous right goal and run on stem prior to left goal choice (R-L, in blue). *C*: an example of place cell activity that only fired on L-R red trials (*bottom*) on 8 trials showing firing field shifting toward the reward location over trials. *A* from Ref. 39, with permission from *Nature Neuroscience*; *B* and *C* from Ref. 34, with permission from *Neuron*.

Dupret and colleagues (39) found that CA1 (but not CA3) place cells shifted their location toward the goal during rewarded navigation and that this reorganization was NMDAR dependent (FIGURE 5*C*). A subsequent study suggested that this movement was greater in deep CA1 pyramids than in superficial ones (44). In contrast, Kobayashi et al. (33) used lateral hypothalamic stimulation as a reward in three tasks (random foraging where randomly chosen locations are rewarded; shuttling between two goals for reward in each; and a goal-directed task where repeated visits to a designated goal location were rewarded) and showed that the majority of place cells (92%) had significant reward correlates only inside the place fields and that reward and place correlates of the neurons did not change between the random foraging and two goal navigation tasks. Only on the third, more complex task did a subset (19%) of place cells shift place fields.

Lee et al. (34) found that during the course of running an automated T-maze alternation task in which the animal was led back to the start after each trial, 70% of place cells fired differently in the stem depending on the animal’s previous and subsequent choice (FIGURE 5, *A* and *B*; the “splitter effect”). These stem-active cells progressively moved along the T toward the goal with experience (FIGURE 5*B*), whereas approximately one-third of cells, those with fields outside the stem, did not change over time. Despite this movement, simultaneously recorded cells maintained their relative topological position. Running the animal on one half of the T configuration without alternation of choices did not result in the forward shift effect. In a candelabra maze task (35) with one start arm and multiple goals visited on different trials, many cells (84%) had multiple fields involving one or more goals. Of these, 61% fired in only one goal and 39% fired in either two or three goals, but there was no evidence that place cells represented goal locations per se. Such a cell would be expected to fire in all four goals and nowhere else: no such cells were seen. Instead, many cells also fired in the start arm, and, similar to Lee et al., 46% of all place fields had different firing rates reflecting the destination on that trial whereas the rest ignored the destination and represented the animal’s actual location in the start arm on all trials. A similar predilection for the start location shows up in other studies as well (40, 42). This has been interpreted as activation of the intended route in the start arm before it is actually run or as conveying information about downstream locations.

**FIGURE 6.**
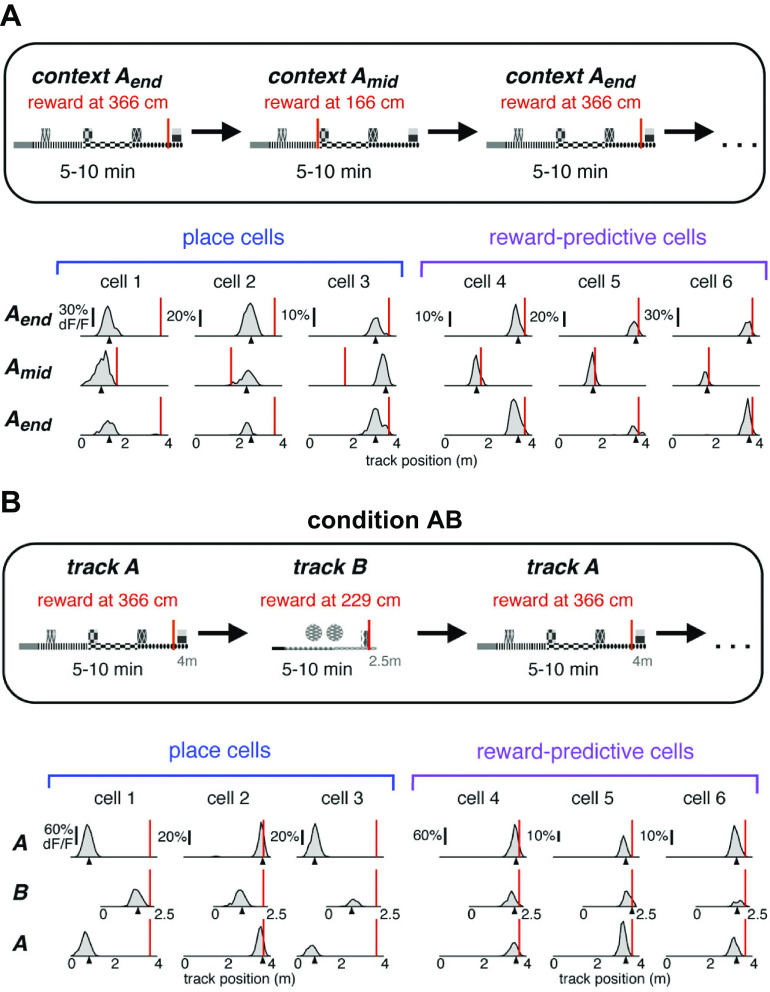
Dedicated population of hippocampal place cells coding for reward location. *A*, *top*: schematic of 1-D virtual reality environment with reward presented either at the end or in the middle of the track. *Bottom*: examples of 3 classic place cells (*cells 1–3*, *bottom left*), which are not affected by shifting the reward location (shown by red line), and reward-predictive cells (*cells 4–6*, *bottom right*), which always maintain their relative position to the reward as it is shifted. *B*: schematic of 2 virtual reality *tracks A* and *B* (*top*) and firing profiles of place and reward-predictive cells on the 2 tracks (*bottom*). From Ref. 45, with permission from *Neuron*.

In general, the majority of existing studies (12 of 13) that reported goal-related firing and that also required the animal to visit more than one goal found evidence that the different goals were represented differently. Only one study (45) reported a small number of cells that fired exclusively at the goal, when it was shifted either to a different location in the same virtual environment (FIGURE 6*A*) or to a totally different virtual environment (FIGURE 6*B*). These “reward-associated cells” comprised 4.2% of cells with fields in both conditions (0.8% of all recorded cells). They might form a small population of place cells with direct inputs from reward circuitry, which then inform other hippocampal cells of reward locations.

Several studies asked whether the value of the goal was represented in place cell firing. That is, if reward in the goal varied, would place cell firing in the goal or elsewhere on the maze represent that change in value (46–49)? Lee et al. (46), using an automated T maze with varying probabilities of reward in the two arms over blocks of trials (signaled by different tones), found that both CA1 (15%) and subicular (11%) cells reflected the animal’s goal choice and its outcome in the current as well as previous trials. In a recent study (49), two goal locations were used, each of which had a different reward value. The rat was required to go to a goal location and remain there for 2 s to elicit a reward delivered elsewhere in the arena. The study failed to find place field clustering at goals but did find increased out-of-field population spiking at the goals occurring when the rat was paused or moving very slowly and only developing after ∼1 s spent at the goal, as had previously been found in one-goal versions of the continuous navigation task (36, 37, 43, 100). This increased out-of-field firing may be related in some studies to sharp wave ripple firing, which has been suggested to be involved in the replay of the previous locations and in the consolidation of the memory of the path to the goal (101). However, it has not been seen in tasks in which animals did not have to wait at the goal (102, 103) or waited there for a shorter delay (38), suggesting that it is this slowing down and waiting in the goal zone that may be the cause of the slight increase in firing and not anything specific about goal location. In general, the amount of waiting time seems to be an important factor, with studies asking the animal to wait 1 s not showing firing whereas those requiring a 2-s wait showing it. McKenzie and colleagues (41) required a 5-s wait and noted that cells started firing on arrival at a goal but decayed in rate thereafter. Forty percent of cells only fired at one location but another 25% fired at more than one, and overall, “It is important to note that cells never fired at all goal locations during WAIT events despite identical behavioral demands” (Ref. 41, p. 10249). The amount of time spent at the reward location cannot be the whole story.

**FIGURE 7.**
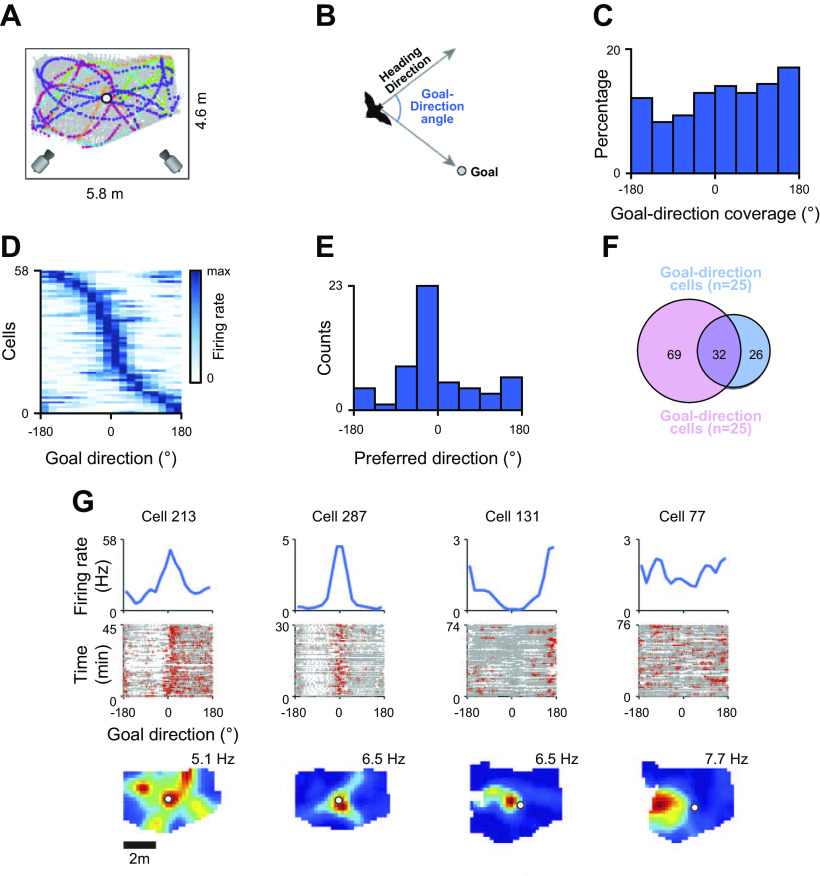
Goal-direction cells in the bat. *A*: schematic of the testing arena used for studying bat goal-direction cells together with typical flight patterns relative to the goal (circle). *B*: polar coordinate system, with heading direction to the goal as the 0° angle. *C*: during flight, bats headed in most directions relative to the goal. *D–F*: 19% of cells had significant directional heading vectors relative to the goal, of which 40% were heading toward the goal and 60% in other directions (*D* and *E*), and 55% also had a place correlate (*F*). *G*: examples of a pure directional cell (*left*), 2 place × directional cells (*center*), and a pure place cell (*right*). From Ref. 51, with permission from *Science*.

Finally, there might be goal-direction and -distance cells, which point in the direction of the goal from various different locations and fire as a function of distance to the goal, respectively. Evidence for CA1 nonplace goal-direction and goal-distance cells was reported in bats (51) (FIGURE 7). These cells were tuned to directions in a polar coordinate framework centered on the goal (FIGURE 7*B*), some of which pointed to the goal (FIGURE 7, *C–G*), and goal-distance cells tuned to specific distances to the goal. Work on rats found weak evidence for goal-direction cells (52) and stronger evidence for goal-distance cells (50). The population activity of these latter cells was directly correlated with the distance from the goal, i.e., cells fired more the farther away the animal was from the goal. Together these two cell types could be used to create a vector to the goal driving the animal’s behavior in that direction.

In an unpublished study on the honeycomb maze, which controls for the behavioral biases introduced during navigation in most situations, we have found that the population of place cells active during navigation formed a vector field pointing toward the goal that shifted when the goal was shifted (J. Ormond and J. O’Keefe, unpublished observations). We can conclude that CA1 place cells either individually or as an ensemble provide vector information pointing to the goal during navigation.

## 9. TIME CELLS

The coding of time as a feature can take several different forms. At its simplest, the time signal can consist of a temporal ordering of events or features and their relative recency, such as whether the experience of a particular smell or sound occurred before or after another. More complex temporal analyses involve the inclusion of metric information such as the duration of an event or the (relative) length of time between events. All of these aspects of temporal encoding can vary in the level of metric scaling involved, and they may or may not share similar mechanisms. Lesion studies as well as single-unit recordings suggest that the hippocampus is important for processing temporal information. However, it still remains unclear to what extent this merely reflects temporal information as a feature generated by other brain regions and embedded in hippocampal locations and contexts (similar to previously described) or as a distinct encoding of a fourth dimension within the hippocampus that is independent of its three-dimensional coding of space (12).

There is clear evidence that scrub jays and rodents can use the time since a visit to a location to control their behavior at that location. Clayton and Dickinson (104) showed that scrub jays could recognize the location and identity of self-cached foods. They trained birds to use the passage of time to decide which foods they had buried to retrieve. They were taught that preferred objects such as worms decayed over time and lost their attractiveness whereas initially less preferred objects such as peanuts maintained their palatability over time. The optimal strategy, therefore, would be to retrieve worms in preference to nuts after short periods since burial and to retrieve nuts after longer periods, and this is what the birds did. Importantly, they also showed that the birds dug in the correct location even if they learned that worms were perishable after they had finished the burying phase of the experiment.

Babb and Crystal (105, 106) designed a similar task for rats and showed that they also can measure and compare relative time intervals between events and associate them with different actions. Rats were trained on an eight-arm radial maze where the daily testing was divided into two phases. During the first “forced choice” phase they were given access to only four randomly selected arms, three of which were baited with regular food pellets and the fourth with a more desirable chocolate pellet. After 0.5 or 4 h they underwent the second “free choice” phase, where all arms were accessible and the four previously unvisited ones were baited with regular food. In addition, after the longer interphase period the chocolate arm was replenished. The results showed that the animals revisited the chocolate arm significantly more often after longer interphase intervals and that this tendency could be reversed if, off-line, chocolate was associated with an unpleasant event such as the sickness resulting from lithium chloride poisoning. It appears that the rats remembered the taste of the chocolate as well as its location (105). More convincingly, training with two flavors in two different arms followed by the devaluation of one by poisoning resulted in only that one being subsequently left unchosen (106).The choice of arms to visit after a delay was not based on the readout from a circadian clock, since the choice could still be made if both choices took place at the same circadian time of day, after intervals of 1 h or 25 h (107). However, it must be noted that at least one alternative interpretation of these results may be that rats were able to encode the recency of the events rather than the relative time intervals per se to solve the task. Importantly, these results were not repeatable when movable unique objects at the ends of the radial maze arms were the predictors of reward instead of the locations themselves (108). In a different set of experiments, the animals could be trained to use their circadian clocks to time-stamp experiences as occurring in the morning or afternoon, and this did not depend on the interval between the feeding experience and subsequent testing (109). This suggests that, similar to scrub jays, rats were able to form episodic-like associations of what-where-when at least within the time frame of a few hours to days.

Another approach to studying the role of time in a spatial context in rodents is to use a modified NOR paradigm where object familiarity is negatively correlated with the relative amount of time since an object was last encountered: higher familiarity leads to less time spent exploring an object (see sect. 6 on object-in-place responses). A typical paradigm (see, e.g., Refs. 110, 111) involves three consecutive exposures to four objects in the same open field box. In the first exposure four copies of the same object A are placed in four different locations; in the second exposure, sometime later, there are four copies of a new object B, some of them in the same locations as the As and some in novel locations. During the third (test) phase the animal is faced with one copy of A and B in old locations (“stationary”) and one copy of each in different locations (“displaced”). The results showed that the animals explored more old stationary objects (A) compared with more recent objects (B), indicating that animals had a knowledge of the relative recency of A and B. The displacement of a familiar object itself is known to result in increased exploration presumably triggering a novelty signal associated with mismatch of an animal’s expectation to find a particular object in a particular place. Interestingly, object displacement reversed the recency-related trend of an animal’s exploratory behavior: now the displaced objects seen more recently (B) were explored for longer compared with displaced old objects (A). This indicates that the temporal recency and spatial object mismatch are not simply added to encode the amount of novelty generated by objects in new locations and their relative recency. One of the difficulties with the approaches described above (at least in relation to rodent work) is that the choices given were binary. As a result, it is difficult to conclude whether this applies to multiple events more generally and, if so, what is the temporal resolution of such encoding.

In another set of experiments, Chiba and colleagues (112) showed that rodents can also tell which location of several was visited earlier. They forced animals to enter arms on an eight-arm maze in a random sequence on each day and then in a test asked them to choose the earlier of two arms visited where the arms were separated by between zero (i.e., sequential) and six intervening arms. The performance of control animals was excellent, showing a gradual increase in performance with larger separation intervals between the choices. Only for pairs of choices experienced in succession was the performance at chance level. Hippocampal animals were severely impaired on this task, being at chance on all pairs except for successive pairs, where the performance was significantly improved. In the case of the latter, presumably a different brain region was used to solve the task (analogous to NOR task performance described above).

These experiments still cannot address some major outstanding questions: do animals (at least rodents) measure the degree of relative recency of various events (A happened before B and B happened before C, because A looks/feels less familiar than B and B is less familiar than C), or, alternatively, can animals genuinely measure intervals (objective or subjective) between events and, if so, what are the time ranges available to them? On a behavioral level, our knowledge about the majority of such “precise” time measurements mostly stems from operant experiments implementing differential reinforcement learning (DRL) or peak timing schedules where after each response, such as a lever press, the animal is required to avoid responding for a predetermined period of time (usually 10–20 s) before responding again. Premature responses reset the clock. Animals with hippocampal damage find this schedule particularly difficult, tending to respond again before the interval is up. In another test of timing ability, the peak procedure, a food pellet is delivered for the first lever press after 20 s of the onset of a stimulus, often auditory, on a fixed-interval 20-s schedule. On 40-s-long nonrewarded probe trials, normal animals typically increase their lever presses to peak at the 20 s time point and then decrease them, demonstrating good duration timing. Both control rats and rats with fimbria-fornix (FFx) lesions (which disconnect the hippocampus from subcortical regions) could perform this task, suggesting that FFx lesion caused no general impairment of a rat’s ability to perceive time intervals (although the perceived time interval might be slightly shortened). The same FFx lesions resulted in profound spatial memory deficits, comparable to hippocampal lesions. However, when a short 5-s gap was introduced halfway through the 20-s stimulus, the controls quickly learned to adapt to it by increasing their estimate of the total length of time by 5 s whereas the FFx animals did not, suggesting that the hippocampus is important for a correct resetting of the timing mechanism, at least when the spatial context is important for the task (113).

Overall, the experimental findings suggest that the hippocampus is important for placing features encountered in a particular spatial context in their temporal order (sequence) and using this information to inform an animal’s future actions. It is also important in setting and resetting the animal’s internal clocks to measure time lapsed in seconds, minutes, hours, and possibly even days (although more work is required to tell how precise the temporal resolution can be). However, the clocks themselves are likely to be located in different, possibly multiple, brain regions located outside the hippocampus (114, 115).

In general, it is difficult to unambiguously interpret the cause of impairments observed in hippocampal animals unless we know more about hippocampal cell activity or until behavioral readouts with a finer indication of an animal’s perception of space and time during the task are developed. The impairment may have arisen because of an animal’s inability to use the information about the temporal order of the events, but equally there might not have been a recollection of the events themselves (i.e., spatial maps of various features and the animal’s actions associated with them); hence their temporal order is unavailable as well, as a secondary consequence.

The cognitive map theory hypothesized that the addition of a linear time signal to the spatial map would provide the basis for an episodic memory system enabling the time-stamping of individual experiences in particular locations (1, 2, 12). There are several ways in which hippocampal pyramidal cells might code for temporal as well as spatial variables. They might increase their activity at a specific time after an event. That is, they might have time fields analogous to place fields and fire after, e.g., 4 s has elapsed since a specific “starting” event. Alternatively, time might be represented by a systematic deviation from the initial coding for a place or an object in a place at the level either of a single cell or of the population. Comparison of this deviated code with the original would act as a measure of time passed. As we shall see, there is evidence for both types of signal in the hippocampus (see Ref. 116 for a recent review).

Eichenbaum and colleagues have reported that hippocampal pyramidal cells signal time within a short series of events as well as the location of those events. In an olfactory recency discrimination (see Ref. 16), Manns et al. (15) found evidence of temporal coding based on systematic alterations in the hippocampal population code representing the sequence of five odor sniffing events. Odor pots changed location across the sequence, permitting detection of firing rate correlates to the odor pot itself, to its temporal sequence in the trial, and to the interaction of these with location. The population of cells showed an average change in absolute firing rates (some increasing and some decreasing) across the session due mostly to rate changes in place cells and less so to the temporal ordering of the sniffing experiences. These changes were correlated with the animal’s ability to perform the task, suggesting that success was due to the association of each odor with the changing place cell activity across the trial and only slightly, if at all, to the temporal sequence in which the odor was experienced.

Another study (55) trained animals on an object/odor go/no-go short-term memory task. Each trial began with the animal sampling one of two objects at the beginning of the stem of a T-maze structure for a few seconds and then entering the middle compartment of the stem for 10 s before being released to sniff at one of two odor pots toward the end of the stem. Each of the objects was matched with an odor pot, and digging at the odor pot of a matching pair yielded a reward; withholding digging to a mismatched pair also yielded a reward farther down the maze. Many cells had place fields in the delay compartment whose field firing was modulated with time passed, forming a spatiotemporal code. For example, a cell might fire during the first few seconds after the animal reached the middle of the compartment, whereas another might fire in the field during the last few seconds. Interestingly, when the length of the delay period was increased, 37% of cells continued to peak during the same period from the start of the delay, whereas 63% shifted their time correlate proportionately. Other cells responded selectively to one of the objects or odors, as might be expected if these were object-in-place cells.

**FIGURE 8.**
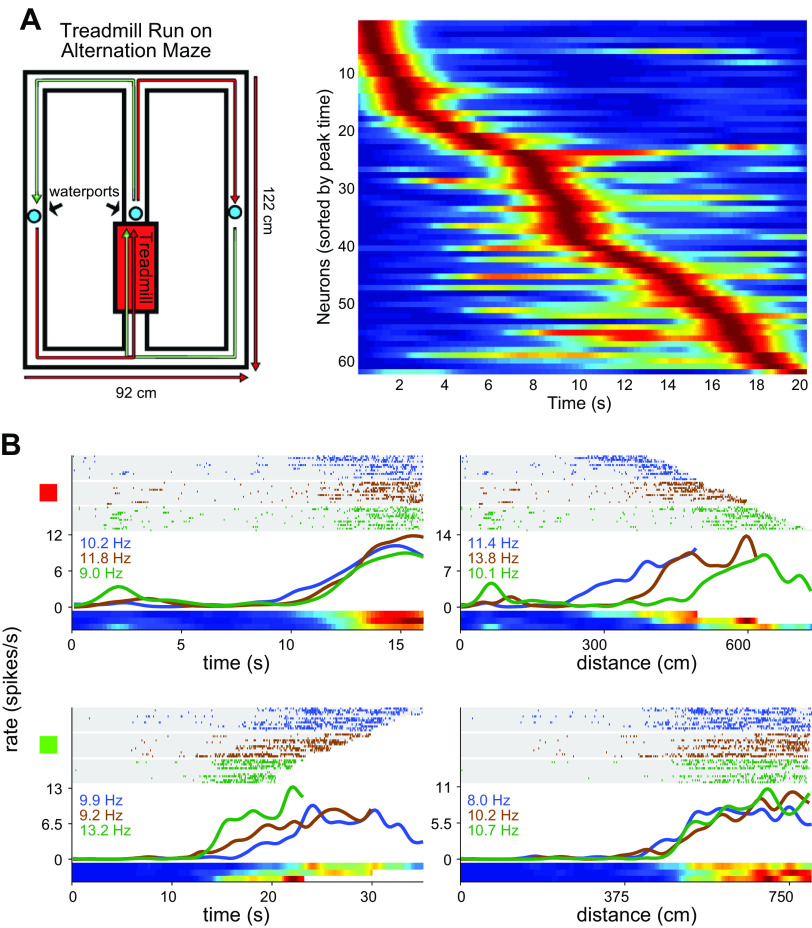
Hippocampal representation of time, short term. *A*: schematic of alternation task with treadmill in stem (*left*) and time cell firing patterns in CA3 as a function of time since treadmill start during treadmill running sessions (*right*). *B*: examples of 2 hippocampal cells, one responding more to time (*top*) and the other to distance run (*bottom*). *Left*: firing rate plotted as a function of time since beginning of run. *Right*: distance run. *A* from Ref. 60, with permission from *Journal of Neuroscience*; *B* from Ref. 57, with permission from *Neuron*.

Other results on short-term time coding are summarized in TABLE 1. Of the six studies listed, only two took context into account and both of these found a strong influence of context. Five of the six studies relied on the technique of forcing the animal to run in position on a wheel or treadmill to look at the firing of pyramidal cells independently of changes in location. In their pioneering study, Czurkó and colleagues (53) recorded while animals explored a small square box or ran on a wheel attached to one side of the box for a water reward. In addition to the usual place cells in the stationary box, a small percentage of cells became active as the animal ran on the wheel and continued to do so as long as the animal continued to run. They concluded that the cells were place cells with fields at the location of the wheel, because rotating the box by 90° either abolished or reduced the firing of the cells on the wheel. Presumably, the wheel had been moved out of the place fields of the cells that were determined in part by the room cues. Alternatively, the firing in the wheel might have been determined at least partially by the head direction system, which would have been altered by the box rotation. In a follow-up study, Pastalkova and colleagues (54) incorporated the wheel into the starting stem of a figure-8 maze that required alternating choices between runs for reward. Each run began with the rat entering the running wheel and running for a short fixed period before being allowed to run down the central stem of the maze and choose left or right. Hippocampal pyramidal cells fired in the wheel as well as on the maze, with different cells active during different times in the wheel running period. The firing pattern of individual “wheel” cells differed between upcoming left and right choice trials and in some animals were predictive of the animal’s choice on that trial. Thus on each trial there was a specific sequence of pyramidal cell firing from the beginning of wheel running through maze choice and on to the reward. Wheel cells had many properties in common with place cells, including showing phase precession. Control recordings were taken when the animals ran in a wheel box similar to that used by Czurkó et al. (see above) and also on a wheel in the animal’s home cage. As in the Czurkó et al. study, cells fired in these wheels but not in such delineated time periods and not in a regular repeatable fashion correlated to the time of running in the wheel. Also similar to the Czurkó et al. study, a different pattern was observed when the animals ran in the opposite direction in the wheel, implicating a substantial role for distal room or head direction cues. Since the animals always ran in the same direction in the alternation figure-8 memory study, a role for room cues can be excluded as a source of variation in the temporal patterns found there. Clearly, hippocampal pyramidal cells can generate sequences of activity during running on wheels independently of currently present environmental cues. Something similar was shown in a virtual reality study (117) in which place cells could be appropriately activated on a linear track after the brief presentation of visual cues at the start of the run that were then removed. In the appropriate experimental conditions, place cells can use some aspect of the animal’s running to update the location of the place cell representation.

Salz and colleagues (60) repeated many of these findings in a slightly different paradigm. They recorded while animals were performing an alternation task on a similar figure-8 maze with a shared central stem containing a treadmill (FIGURE 8*A*, *left*). Twenty-seven percent of CA3 cells and 30% of CA1 cells increased their activity on the treadmill, and their peaks ranged from the beginning of running to the end (FIGURE 8*A*, *right*). Interestingly, the time period during which the cells fired lengthened with increasing time during the run, resulting in decreased temporal resolution. Roughly the same percentage of “time” cells were recorded when the working memory component of the task was removed by using only one half of the figure-8 structure. In an important dissociation study, Kraus et al. (57) forced the animal to run at different speeds on the figure-8 treadmill, potentially allowing dissociation of distance run from time spent running. Most neurons were influenced to varying extents by both variables, but there were cells at the extreme ends of the distribution that were influenced entirely by one of these variables (FIGURE 8*B*). A similar finding was reported by Villette et al. (61) and Haimerl et al. (62), who imaged CA1 pyramidal cells as mice ran or stood still on a nonmotorized treadmill in a featureless environment in the dark. During spontaneous running, they observed spontaneous bursts of firing in populations of 5% of the cells referred to as “sequences of neural activation,” which represented the distance run (50% of sessions, 14/28), the duration of running (11%, 3/28), or a mixture of both (39%, 11/28); overall, the majority represented distance significantly stronger than time (61, 62). Animals tended to spontaneously run in bursts of distance lengths that were multiples of the neural activation sequence lengths, prompting Villette and colleagues to suggest that each population sequence encoded distance traveled in discrete “distance units” (61). It should also be noted that sequences of neural activations were only present when the animal was running and not when it was standing still, suggesting that they may be important for integrating speed rather than measuring time intervals. This view was strengthened by the observation that short pauses in running during a sequence did not disrupt the sequence progression, suggesting that it was more related to distance than time. Importantly, the bursts of activity in the same cells recorded on two consecutive days could switch from one mode of representation to another, showing that the same circuits could provide either spatial or temporal information. Modeling the dynamics of the hippocampal circuitry and examining the variables that produced the switch suggested that a generalized input such as an increase in the power in the theta band could switch the network from the duration to the distance mode (62). One interpretation is that a decrease in the running speed information provided by the septal-generated theta signal removes the input necessary for the hippocampal network to calculate distances traveled by taking running speed into account. This idea that it is the sequential behavior of hippocampal pyramidal cells, whether representing temporal or spatial variables or both, that is important echoes a position advocated by Buzsáki and Tingley (7). Before we accept the simple interpretation that there are “time” cells that have as their primary or sole correlate the time after an event, we might want to see what these same cells do on running wheels located in different parts of the maze or in different locations in the room, to see whether the time cells are actually time-in-place cells analogous to the feature-in-place cells described elsewhere in this article. To our knowledge, this experiment has not yet been carried out. Furthermore, animals trained to run on treadmills may not be running in the same way that they would over immobile terrains. For example, in early studies of theta activity on treadmills, it was reported that theta frequency was maximum at the beginning of a run but settled down to an asymptote frequency and did not correlate with running speed (118). This is at odds with more recent findings from animals running on linear tracks without a treadmill, where the theta frequency correlates with speed (see, e.g., Ref. 119).

Another way in which a time code might be instantiated is by rate remapping of place cells across time. That is, place fields could stay in the same location over time but firing rates slowly diverge from the original in a probabilistic way, giving rise to a temporal signal at the population level. Several studies support this possibility.

**FIGURE 9.**
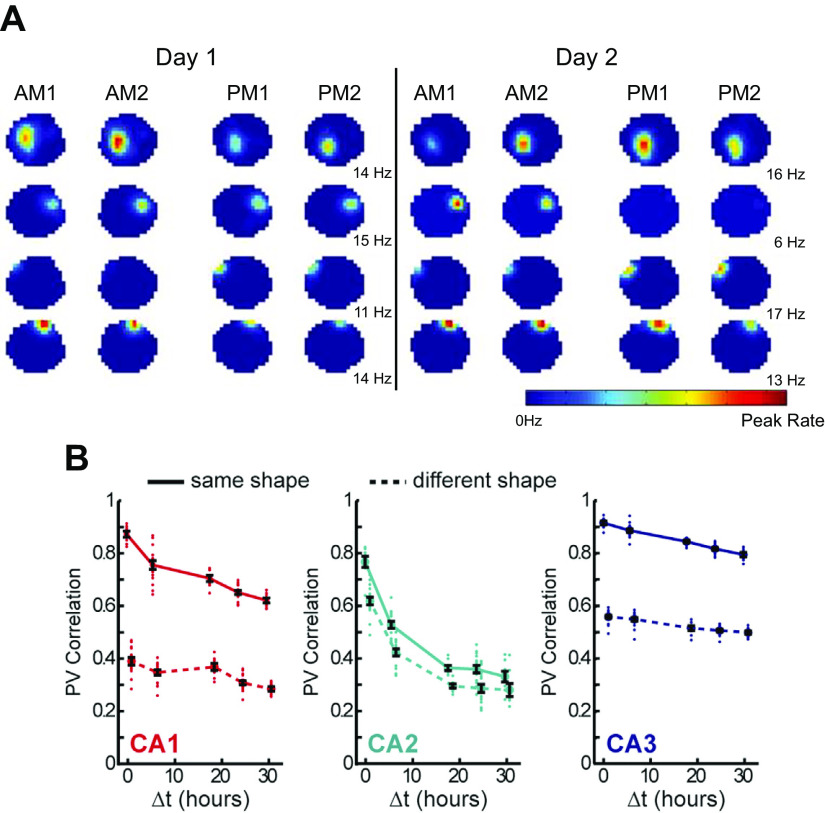
Hippocampal representation of time, long term. *A*: changes in firing rates of 4 CA1 place cells with successive experiences of the same environment over 2 days. Note the variability in rates but not field location over time. *B*: change in population vector (PV) correlations with time in the same (solid lines) and different (dashed lines) environments in the CA1, CA2, and CA3 fields. Note the larger slope in CA1 vs. that in CA3 in the same boxes, which might be used as a time signal. Note the different temporal profile in the CA2 population. From Ref. 56, with permission from *Proceedings of the National Academy of Sciences of the United States of America*, and from Ref. 59, with permission from *Neuron*.

In the first, by Mankin and colleagues (56), CA1/3 place cells were recorded in the same box for a total of eight recording sessions of 10 min each over 2¼ days (FIGURE 9*A*). CA1 place cell population firing rates deviated systematically from those of the first experience as a monotonic function of time (FIGURE 9*B*); the deviations in the CA3 population recorded at the same times were statistically smaller. The changes were slightly greater in both CA regions when two different boxes were used, but the absolute difference between them was maintained. However, when changes in firing rates within each session were examined, a different pattern emerged. Now it was the CA3 cells that changed rates to a greater extent than those in CA1 (not shown). Comparison of firing rates over time between the CA1 and CA3 populations provides both short-term (ΔCA3 > ΔCA1) and long-term (ΔCA3 < ΔCA1) temporal modulation of the place field code. CA2 cells showed greater changes over time than either CA1 or CA3 (59).

Corroboration of the long-term temporal code in CA1 comes from a study that recorded calcium transients from large numbers of hippocampal pyramidal cells for up to 45 days (58). Place fields were stable but disappeared and reappeared on a random basis, causing the cell population pattern to deviate from the original, similarly to the Mankin et al. experiment (56) described above. The deviation from the original pattern continued for as long as 30 days, reaching quite high levels despite which there was enough similarity between any two patterns to enable a decoder to use the pattern recorded on the first occasion to identify the animal’s position on the second. This means that in addition to providing a temporal code for the linear passage of time as the place cell patterns deviate from each other, there is still enough stability in their firing to reliably reidentify the animal’s position across considerable periods of time. We can expect this spatial code to be much more reproducible in CA3 if the pattern shown in the Mankin et al. experiment continues for the longer periods used in the Ziv et al. experiment (58). In a subsequent study, Rubin and colleagues (120) trained mice in two distinct runways located in different parts of the same laboratory. Both population codes drifted away from the originals at roughly the same rates, and temporal decoders trained on one set of pyramidal cells could predict elapsed time on the other maze with a significant accuracy of 65% (compared with 98% within tracks). This finding can be taken as some evidence for a cross-environment temporal code. It cannot be ruled out, however, that the temporal code for the two mazes was embedded within a roomwide spatial context.

Another important example of encoding the temporal sequence of distinct events within a single overall experience that can be generalized across multiple analogous experiences was recently reported by Sun and colleagues (63). In this experiment the authors used one-photon microscopy to image calcium transients in a large number of CA1 pyramidal cells from mice running on a square-shaped linear track. A single running session comprised four laps, and animals were food-rewarded in the same corner of the track at the beginning of each session. The animals completed 15–20 sessions in succession. Although 72% of the CA1 pyramidal cells had place fields, they also noted that for 31% of these cells [called event-specific rate remapping (ESR) cells], the firing level within the place field varied with the number of laps after reward, for example, firing on the second, but not other, laps. Most of ESR cells fired on the first lap (∼50%), but other laps were also represented (∼16% of ESR cells for each subsequent lap). Importantly, they showed that these event-specific rate remapping responses could be dissociated from the location of the place fields. Switching to a circular maze caused the place fields to remap, but 38% of the cells with strong event-related responses continued to mark the same lap. Conversely, they were able to change the preferred lap by increasing the number of laps run before reward or by blocking the inputs from the mEC. This latter caused the preferred lap to shift but left the place field unaltered. Alterations of time elapsed or distance run after the reward did not affect the preferred lap. The effect increased with experience, and, conversely, rewarding the animal on each lap greatly reduced the effect (to 9% of cells; and in this case every lap was equally well represented), showing the importance of the lap-polarizing rewarding event. It is unfortunate that they did not alter the amount of reward or try a different polarizing event such as interrupting the animal’s progress every fourth lap to demonstrate the generality of the effect. This experiment would seem to demonstrate that some aspect of the number of laps subsequent to or before the feeding experience was being encoded in the pyramidal cells independent of the place code. However, it must be noted that on average only ∼4.6% of place cells per animal (8 of 179 of corecorded place cells) represented the order of each lap beyond the first lap (i.e., 2nd, 3rd, and 4th laps). Taken together with the observation that in the majority of given examples these lap responses were not confined to just a single lap (i.e., they were nonbinary and often present on other laps, only being strongest on a particular lap on average), this warrants more careful investigation of whether ESR cells are really used to encode the order of the “events” beyond the first lap. The differential firing during the rewarded event (i.e., the 1st lap) may represent the presence of the reward rather than any temporal order per se.

In the absence of evidence that animals use these codes to guide their behavior, we should be cautious of overinterpreting them as evidence for temporal coding. An alternative possibility is that the ongoing changes of place cell responses during different visits may reflect the flexibility of the hippocampal code to adopt to constantly changing experience (each visit is different even if it occurs in the same environment). Hence it may allow the animal to distinguish between different events in the same spatial context, but whether the animal can actually use it to assess temporal relations between these events remains to be seen. As described above, Manns et al. (15) as well as Allen et al. (18) found evidence of temporal coding based on systematic alterations in the locational population code representing the sequence of five odor sniffing events, and these were correlated with the animal’s ability to perform the task, suggesting that this form of spatiotemporal code could be useful in solving temporal sequence problems.

At this point it is clear that the hippocampus incorporates a representation of time of different durations. Whether this is independent of the spatial mapping function is not clear, since in general the relevant experiments have not been done. In only two experiments (63, 120) is there some evidence that time can be represented independently and not as a variable within the spatial framework comparable to other features. The independence of hippocampal temporal coding from spatial coding is an important question that needs more carefully controlled behavioral physiological experiments.

## 10. SOME REFLECTIONS ON OBJECT/FEATURE-IN-PLACE CELLS AND SUGGESTIONS FOR FUTURE EXPERIMENTS

Before concluding, let us make some general reflections on this review of feature-in-place experiments. The first is that it is not always obvious which strategies animals actually use to solve a problem, even though the experiment has been designed with a particular solution in mind. It is necessary to demonstrate experimentally by probe trials or transfer tests that animals are using a particular solution. In the Manns et al. odor recency study (15), it seems obvious that animals should use the strategy of comparing the relative strengths of recently experienced odors to solve the task, but they did not, instead preferring to use the recency of an odor in a specific place. Importantly, these animals could correctly perform a recency discrimination between two experienced odors independently of place if tested in locations different from the original ones despite the fact that no hippocampal cells underpinned this direct coding of an odor experience independent of place. The information must have been contained in the activity of extrahippocampal cells but not used when placed in competition with the spatially based information. This probably explains results where animals are originally taught a task and lose that ability after hippocampal lesions but can learn to do the task as quickly as control animals if the lesions are made before training. In the first case the hippocampal code overshadows the nonhippocampal information, whereas in the second the hippocampal information is not available. This idea that animals entertain several different hypotheses to the solution of a problem was originally introduced by Krechevsky (121) and adopted by O’Keefe and Nadel (1). A good example of the experimental verification of this idea is the work by Packard and McGaugh (122), who showed that a latent alternative strategy for solving a problem could be uncovered by pharmacologically blocking the brain region supporting the dominant strategy.

The second point to make is that, given the claim by the cognitive map theory that the primary function of the hippocampus is to store information about the location of objects and features and only secondarily about the objects/features themselves, it is incumbent on experiments seeking to demonstrate exclusively nonspatial inputs to the hippocampus to exclude the possibility that these inputs are secondary to feature-in-place representations. The Sun et al. experiment (63) on event-specific rate remapping responses is an important exception. It will be interesting to see if mice can be taught to signal that they know that they are on a particular lap in this or similar experiments. An important corollary of the possibility that many of the simple feature responses are actually feature-in-place responses is that the numbers given in this review for the percentage of cells that respond to a particular stimulus are clearly underestimates, since there may be many hippocampal cells that respond to, e.g., an odor in an environment other than the one tested in a particular experiment. Ideally, to get a clear idea of how many hippocampal cells carry information about a particular feature such as a smell or a tone, it will be necessary to test that feature in multiple different environments.

Third, in some experiments the stimuli used or observed have been continuous physical variables such as the frequency of a tone, textures of different roughness, or intervals of time, and this has led some authors, for example, Aronov et al. (5), to speculate that perhaps the hippocampus is a more general representational structure coding for any continuous physical variable, with space being merely a good example. But a moment’s reflection on the full spectrum of stimuli that can be represented as features in place should make it clear that these are not limited to continuous variables but can also include discrete ones. For example, as reported above, different tastes or odors that are not representable on a continuous physical scale are represented as features in place in the hippocampus. It would appear that the hippocampus can represent any feature that can be localized to a particular place as having been experienced in that place.

The fourth point to make relates to the complete absence of information about whether and how hippocampal cells might represent two different types of features occurring in the same place. Although different studies have looked at the distribution of cells that respond to, for example, four different tastes in multiple places (19) or to 20 different odors in several places (16), there are no studies that have looked at the relationship between these two different feature-in-place groups of cells. Are these distributions totally different, or is there an overlap between them? In the latter case, do the cells respond differently to the two features, perhaps with different firing rates? We might speculate that they might do so by rate remapping, with firing rate higher to one of the features than the other. Another possibility is that they fire on different phases of the theta or gamma cycle. We might further speculate that the occurrence of two different features in the same place might serve to bind them together. The animal might know that a tone and a smell went together because they both occurred in the same place.

The fifth point is that the responsiveness of hippocampal pyramidal cells to features in a place is often stronger or solely dependent on the animal’s performing in a task that engages its attention. For example, in the Aronov et al. (5) and Itskov et al. (22) experiments the cells did not respond to the sensory stimuli when they were presented under conditions where attention was not required. This also appears to be true in the representation of the location of other animals. Although it is clear that simple place responses in pyramidal cells are stronger when attention is required, e.g., in spatial discriminations, there is still a strong place response even when the animal is attending to something else, as, for example, in the Muller foraging task. It is also clear that learning about features in a place increases the number of cells representing that association (16, 17). Perhaps the feature-in-place correlate of hippocampal pyramidal cells is more dependent on attention or learning than the simple place correlate.

Sixth, and finally, it should be noted that the spatial context effect can manifest itself on different environmental scales ranging from locations within a single box to different boxes or different rooms. The spatial scales of place cells vary along the long axes of hippocampus, getting larger as one moves more ventral in the structure (95, 123). We might expect that place discriminations confined to small spaces such as odor location within a box might be dependent more on the dorsal hippocampal cells whereas discriminations involving locations across different boxes or different parts of the room might be found in the ventral hippocampus (97). One bioassay for whether a new location represents a context shift might be to see whether simultaneously recorded place cells remap.

## 11. CONCLUSIONS

We can conclude that, in general, hippocampal pyramidal cells represent stimuli such as smells, tastes, tones, objects, and conspecifics or events such as the sequence in which odors were experienced within a spatial framework, as modulations of place field or spatial context firing. From a total of 65 studies that looked at nonspatial coding in hippocampal pyramidal cells, 27 did not change the location in which the event occurred or the stimulus was delivered. Of the 38 that did test location dependence, 33 (87%) found a strong or complete dependence. This modulation often takes the form of a rate change but in some cases can result in remapping of the place field. The clear exceptions to this rule are the experiments by Wood et al. (14), Gauthier and Tank (45), and MacDonald et al. (55), which found small numbers of nonspatial responses (8%, 5%, and 11% of cells, respectively) in addition to the large numbers of spatially dependent ones [e.g., 73% in the MacDonald et al. (55) experiment], and the recent Sun et al. (63) experiment, which indicates that the representation of lap number within a sequence may be independent of the place field representation in the same cell. It is still an open question as to whether ordinal sequences and temporal events more generally can be represented by the hippocampus independently of place or whether this latter finding is specific to some aspect of the behavioral paradigm used. More experiments of this type might help to decide.

The representation of objects or conspecifics within the mapping system can often occur independently of whether the animal is involved in a task requiring the coding of object identity or presence. For example, it occurred in the incidental learning of objects and their location in the Manns and Eichenbaum task (26) or in the presence of conspecifics in the von Heimendahl et al. (27), Alexander et al. (29), and Zynyuk et al. (28) studies. On the other hand, engagement in the task seems important in other studies (5, 31).The hippocampal system is capable of mapping discrete categorical entities such as the presence or absence of a tone signaling eyelid shock and the experience of different tastes or odors as well as representing continuous variables such as the frequency of a tone or the order of events as required in recency discriminations. That is, it is capable of representing events embedded in spatial contexts on categorical and ordinal scales. Whether it is also capable of representing events on interval and absolute scales has yet to be tested. This would require quite a complicated test such as choosing an object or cue that was repeated with a shorter interval over one repeated with a longer interval, irrespective of the order of presentations.

## GRANTS

J.O. is a Wellcome Trust Principal Research Fellow (203020/Z/16/Z) and is supported by the Sainsbury Wellcome Centre Core Grant from the Gatsby Charitable Foundation and Wellcome Trust (090843/F/09/Z). J.K. is a Wellcome Trust/Royal Society Sir Henry Dale Fellow (206682/Z/17/Z) and is supported by Dementia Research Institute (DRICAMKRUPIC18/19), Isaac Newton Trust/Wellcome Trust ISSF/University of Cambridge Joint Research Grant, Kavli Foundation Dream Team project (RG93383), Isaac Newton Trust [17.37(t)], and NVIDIA Corporation.

## DISCLOSURES

No conflicts of interest, financial or otherwise, are declared by the authors.

## AUTHOR CONTRIBUTIONS

J.O. and J.K. analyzed data; drafted manuscript; edited and revised manuscript; and approved final version of manuscript.
